# Bibliometric and knowledge-map analysis of research on robot-assisted vascular interventional surgery (2015–2025)

**DOI:** 10.1007/s11701-026-03947-9

**Published:** 2026-09-12

**Authors:** Yanwen Liu, Shanshan Li, Chenggang Liu

**Affiliations:** 1 Nanjing University of Aeronautics and Astronautics, Jiangjun Road No.29, Jiangning District, Nanjing City, 211106 Jiangsu Province China; 2 School of Basic Medical Sciences, Heilongjiang University of Chinese Medicine, 24 Heping Road, Harbin, 150040 China

**Keywords:** Robot-assisted vascular interventional surgery, Bibliometrics, Knowledge mapping, Embodied intelligence, Autonomous navigation

## Abstract

Vascular interventional procedures are minimally invasive but remain highly dependent on operator expertise and are associated with occupational radiation exposure and technically demanding catheter and guidewire manipulation. By integrating precision motion control, force sensing, haptic feedback, image-guided navigation, and teleoperation, robot-assisted vascular interventional surgery (RVIS) has emerged as an interdisciplinary field spanning robotics, biomedical engineering, and interventional medicine. However, its global research landscape, intellectual base, research hotspots, and thematic evolution remain insufficiently characterized. This study therefore systematically mapped the knowledge structure and research trends of RVIS from 2015 to 2025. The Web of Science Core Collection (WoSCC) and Scopus were searched for relevant English-language articles and reviews published between 2015 and 2025. After screening according to uniform eligibility criteria, 373 WoSCC records and 493 Scopus records were included. VOSviewer, CiteSpace, and Bibliometrix/Biblioshiny were used to analyze annual publication trends, country and institutional collaboration, authors, journals, co-citation relationships, highly cited publications, citation bursts, and keyword networks separately for the two databases. After cross-database deduplication, an English-abstract corpus was constructed from 541 unique publications, of which 524 met the eligibility requirements for latent Dirichlet allocation (LDA) modeling. The model fitted to the merged corpus served as the primary integrative analysis, while database-source sensitivity analyses were conducted in the WoSCC-only and Scopus-only corpora using the same five-topic configuration. Multiple testing in the temporal topic-trend analyses was controlled using the Benjamini–Hochberg procedure. Annual publication output in both databases showed an overall upward, albeit fluctuating, trend and remained relatively high during the later years of the study period. China and the United States were the two most prominent contributors to the field. In the corresponding-author country analysis, China ranked first and the United States second in WoSCC, whereas China and the United States ranked jointly first in Scopus. Analyses of core journals, authors, and co-citation networks indicated that the intellectual base of the field was jointly shaped by robotics and control technologies, biomedical engineering, computer-assisted surgery, and cardiovascular and neurovascular interventions. Highly cited publications, citation bursts, and keyword networks primarily focused on magnetically actuated guidewires and soft robots, endovascular catheter control, intelligent navigation, force and haptic feedback, and robot-assisted percutaneous coronary intervention. LDA identified five topics, of which remote image-guided platforms had the highest overall topic prevalence. After correction for multiple testing, the topic of magnetically actuated soft robots and microrobots showed a significant upward trend in the merged corpus. The single-database sensitivity analyses demonstrated moderate reproducibility of the principal topics, with the annual trend for magnetically actuated soft robots and microrobots showing a consistent direction across all three corpora. Research activity in RVIS increased overall between 2015 and 2025, accompanied by the emergence of an interconnected knowledge structure spanning engineering technologies and clinical interventional medicine. Catheter and guidewire manipulation, remote intervention, intelligent navigation, force sensing, and haptic feedback represent major research directions. The relative research attention devoted to magnetically actuated soft robots and microrobots increased consistently across all three LDA corpora. These findings reflect changes in publication output and research attention and should not be interpreted as direct evidence of technological maturity, clinical adoption, or patient benefit.

**Supplementary Information:**

The online version contains supplementary material available at https://doi.org/10.1007/s11701-026-03947-9.

## Introduction

Robot-assisted vascular interventional surgery (RVIS) represents a convergence of minimally invasive medicine and precision mechanical engineering and is intended to address several persistent limitations of conventional interventional procedures, including prolonged occupational exposure to ionizing radiation, operator fatigue, and physiological hand tremor [1]. Through a master–slave control architecture, RVIS systems translate the operator’s hand movements into precise intravascular movements of catheters and guidewires. This approach improves the operator’s working environment while enhancing procedural precision and safety through tremor suppression and motion scaling [2]. Beyond mechanical design, current research also encompasses human–robot interaction, haptic feedback, and artificial intelligence (AI)-enabled navigation and decision support. Although these studies demonstrate an expansion of the field’s research scope, they do not establish that RVIS has completed a transition from teleoperation to semiautonomous or fully autonomous operation [3, 4]. With respect to master–slave control systems and mechanical design, vascular interventional robots require considerable flexibility and compatibility to accommodate interventional devices of different diameters and materials. Existing distal manipulators generally employ multi-degree-of-freedom designs capable of reproducing the complex insertion, retraction, and rotation of guidewires and catheters performed manually by interventionalists [5, 6]. To address inaccurate motion transmission and insufficient operational stability in conventional master–slave control systems, researchers have developed systems with isomorphic master–slave configurations, making master-side manipulation more consistent with conventional operating practices and potentially reducing the learning curve [7]. In addition, novel actuation mechanisms, including helical magnetic robots, have been proposed to improve navigation through tortuous vascular pathways encountered in procedures such as neurovascular interventions [8]. The absence of haptic feedback has long been regarded as a major barrier to the broader clinical adoption of vascular interventional robots. During conventional manual procedures, experienced interventionalists use fingertip perception of the contact force between the guidewire and the vessel wall to assess device position and avoid vascular perforation. By contrast, most existing robotic systems rely predominantly on visual feedback, resulting in diminished haptic perception [9]. Recent studies have therefore sought to restore this essential sensory channel by integrating tactile sensors and force-feedback algorithms. Experimental findings indicate that visual force feedback and vibrotactile feedback can enable more controlled application of force during delicate manipulation and may thereby reduce the risk of tissue injury [10, 11]. Nevertheless, signal latency, resolution, and synchronization with imaging systems remain critical performance parameters requiring further optimization [12]. With the increasing integration of AI, vascular interventional robots are gradually acquiring autonomous navigation and decision-making capabilities. Conventional guidewire navigation depends heavily on operator experience and is associated with considerable uncertainty in complex vascular anatomies. Recent studies have used deep learning and reinforcement learning to train intelligent agents to acquire procedural decision-making skills in virtual environments and have subsequently evaluated the transfer of these learned strategies to physical robotic systems [4]. Active suppression of hazardous behaviors has also been incorporated into robotic systems to monitor operational states in real time and automatically identify and prevent actions that could cause vascular injury, thereby providing an additional algorithmic safeguard for procedural safety [13]. These developments offer potential technical pathways for reducing operators’ cognitive workload and expanding the feasibility of remote intervention, although their clinical effectiveness requires further validation [14].

Despite continued technological advances, RVIS still faces considerable barriers to clinical translation. Bibliometrics provides a quantitative approach to analyzing scientific literature and can systematically characterize the research hotspots, intellectual structure, and thematic evolution of a field. A previous bibliometric study examined robot-assisted vascular surgery [15]; however, its scope encompassed both open surgical robotic systems and vascular interventional robots and did not investigate RVIS as a distinct subspecialty. Meanwhile, previous research on robotic surgery and AI in vascular surgery has generally focused on broad surgical applications, intelligent imaging, or digital health. Systematic reviews of safe human–robot collaboration have primarily addressed the application of AI in risk-assessment methods and issues related to safety, reliability, and standardization [16], without specifically examining robotic catheter and guidewire manipulation in endovascular settings. Consequently, the intellectual structure jointly formed by engineering research and clinical interventional medicine, as well as its thematic evolution within the RVIS field, has not yet been systematically characterized. Accordingly, this study used the Web of Science Core Collection (WoSCC) and Scopus as its data sources and applied an explicit operational definition and uniform screening criteria to delineate the target literature. Bibliometric analysis, science mapping, and topic modeling were performed to characterize the principal research contributors, collaboration networks, intellectual base, research hotspots, and temporal changes in the field from 2015 to 2025, thereby providing a structured account of the interdisciplinary landscape and research development of RVIS.

## Methodology

### Data sources, search strategy, and study scope

The Web of Science Core Collection (WoSCC) and Scopus databases were searched for publications on robot-assisted vascular interventional surgery (RVIS) published between 2015 and 2025. The publication period was restricted to January 1, 2015, through December 31, 2025. A topic search was performed in WoSCC, whereas the title, abstract, and keyword fields were searched in Scopus. The search terms comprised two principal concept groups—robotic technologies and vascular interventions—and were adapted to the field-specific syntax of each database. Eligible document types were restricted to articles and reviews published in English. The complete search strategies and search execution dates are provided in the Supplementary Material.

### Literature screening, cross-database deduplication, and data cleaning

For this study, RVIS was operationally defined as an intravascular or endoluminal interventional procedure involving a catheter, guidewire, or actively navigated device in which a robotic system directly participates in or controls intravascular device manipulation, navigation, positioning, or therapeutic delivery. Clinical, preclinical, engineering, and review studies focusing on such systems were eligible for inclusion. Although the relevant literature encompassed diverse clinical settings and technological platforms—including coronary, neurovascular, aortic, and peripheral vascular interventions; transcatheter procedures; and magnetically actuated intravascular robots—all included studies shared the defining characteristic that a robotic system directly participated in intravascular device manipulation or navigation. These studies were therefore regarded as distinct technological and application subdomains within a common operational boundary of RVIS.

**Fig. 1:**
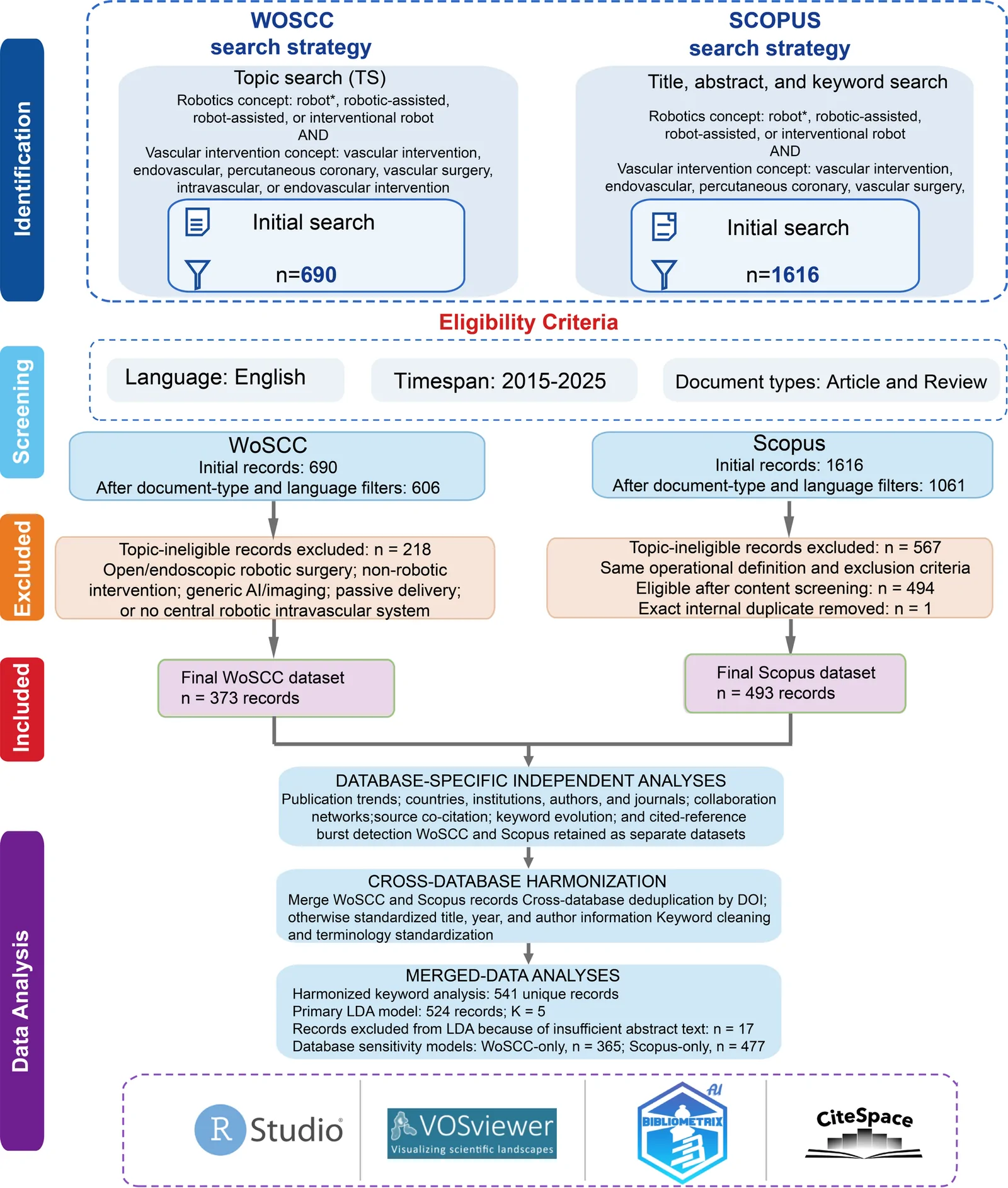
Workflow of the data search strategy

Studies were excluded if they involved open, laparoscopic, or thoracoscopic robotic vascular surgery; coronary artery bypass grafting without robotic intravascular manipulation; nonrobotic vascular interventions; general AI-based image analysis; simulation studies without a core robotic interventional system; passive nanomedicine delivery; or other non-intravascular procedures. Records from both databases underwent title and abstract screening according to the same eligibility criteria. Ultimately, 373 WoSCC records and 493 Scopus records were included. Detailed screening procedures, the numbers excluded at each stage, and the decision rules applied to borderline cases are provided in the Supplementary Material. The literature screening process is illustrated in Fig. 1.

Bibliographic records from the two databases were cleaned separately. DOI formats, title spacing, punctuation in author names, journal names, institutional names, and country or region names were standardized. Within-database duplicates were first identified using standardized DOIs. For records without a DOI, the title, publication year, and first author were jointly examined, and the record containing the most complete bibliographic information was retained. Author names were disambiguated using full names, institutional affiliations, coauthorship relationships, and available ORCID information. Name variants were merged only when the surname, full given name, and middle-name information were compatible. Records with clear conflicts in full names, affiliations, or collaboration patterns were retained as distinct authors. Abbreviated and full institutional names, including expressions referring to affiliated hospitals, were standardized. Keywords were sequentially standardized for capitalization, spacing, and hyphenation; singular and plural forms, spelling variants, and semantically equivalent terms were merged; and duplicate keywords within individual records were removed. Representative standardization examples and detailed data-cleaning rules are provided in the Supplementary Material.

### Descriptive bibliometric and collaboration network analyses

Descriptive analyses of annual publication output, countries, institutions, authors, journals, and citation indicators were conducted using R version 4.6.1 and Bibliometrix/Biblioshiny. Annual publication trends were presented solely as annual and cumulative publication counts. No polynomial curve fitting was performed, and publication trends were not used to predict future growth or infer technological maturity, stages of innovation diffusion, or levels of clinical adoption.

Country participation was assessed using full counting. A country received one publication credit whenever at least one author affiliation from that country appeared in a publication; consequently, the sum of country-level publication counts could exceed the total number of included publications. Corresponding-author country analysis was conducted separately to calculate single-country publications (SCPs) and multiple-country publications (MCPs). Publications involving only one country were classified as SCPs, whereas those involving two or more countries were classified as MCPs. The MCP proportion was used to describe the relative extent of international collaboration associated with each corresponding-author country.

Fractional counting was used to reduce the inflation of author- and institution-level rankings caused by large collaborative teams. For publications involving multiple authors or institutions, publication and citation credits were divided according to the corresponding number of authors or institutions. Author and institutional collaboration networks were visualized using full counting to represent collaborative relationships; these networks were not used as substitutes for fractional contribution rankings. Author rankings therefore represent relative publication contributions and citation attention within the corpus included in this study and should not be interpreted as direct measures of an author’s overall scholarly influence.

Total citation counts were calculated separately using the citation data recorded in WoSCC and Scopus at the time of retrieval. To reduce citation accumulation bias associated with publication year, total citations per year (TC/year) were also calculated by dividing the total citation count by the number of years from the publication year through the database search year, with the publication year counted as the first year. Journal impact factors were based on the 2025 Journal Impact Factor reported in Clarivate’s Journal Citation Reports. The data source and access date are specified in the Supplementary Material.

VOSviewer version 1.6.20 was used to construct country collaboration, author collaboration, keyword co-occurrence, and source-journal co-citation networks. Networks were normalized using the association-strength method. Node size represented publication output, keyword occurrence frequency, or co-citation frequency, as appropriate. Links denoted collaboration, co-occurrence, or co-citation relationships, and link thickness represented the strength of these relationships. Different colors indicated network clusters identified by the software algorithm. Minimum inclusion thresholds, numbers of included nodes, and other parameters for each network are reported in the Supplementary Material.

### Journal, citation, and keyword evolution analyses

Source-journal distributions and journal co-citation analyses were conducted separately for the WoSCC and Scopus datasets. Source-journal analysis was used to identify the principal publication outlets in the field, whereas journal co-citation analysis was used to characterize its intellectual base. Because WoSCC and Scopus differ in journal coverage, reference indexing, and citation-counting mechanisms, comparisons between the two databases were used primarily to describe qualitative similarities and structural differences in the research landscape rather than as a formal test of cross-database robustness.

CiteSpace version 6.4.R1 was used to analyze citation bursts of cited references and the temporal evolution of keywords. The time span was set to 2015–2025, with one-year time slices and the Top N set to 50 for each slice. For burst detection, γ was set to 1 and the minimum burst duration to two years. Pathfinder and pruning sliced networks were applied for network pruning. Citation-burst analysis was used to identify studies that experienced rapidly increasing citation attention during specific periods, whereas keyword temporal analysis was used to characterize the emergence and persistence of research topics over the study period. Burst results indicate temporally concentrated changes in citation attention and do not directly represent technological maturity or stages of clinical adoption.

Cross-database comparisons of keyword frequencies were restricted to author-keyword fields from WoSCC and Scopus to improve field-level comparability. Database-specific keyword networks were constructed using the keyword fields available in each database. Because Scopus index keywords may be influenced by database-specific controlled indexing practices, networks involving index keywords were used only to describe the internal thematic structure of the Scopus corpus and were not directly compared quantitatively with WoSCC author-keyword rankings. Keyword sources, cleaning rules, and inclusion thresholds for each database are detailed in the Supplementary Material.

### Cross-database data integration and deduplication

After independent screening, 373 WoSCC records and 493 Scopus records were retained. Bibliometric analyses of countries, institutions, authors, journals, co-citation relationships, highly cited publications, and keyword networks were conducted separately for the two databases. For cross-database integration, DOI formats were first standardized. Records without DOIs were subsequently matched and manually verified using standardized titles, publication years, and first-author information. A total of 325 pairs of duplicate records were identified across the two databases: 317 pairs were matched exactly by standardized DOI, and eight pairs were matched exactly by standardized title. Auxiliary fuzzy matching identified no additional duplicate records. The resulting dataset comprised 541 unique publications, including 48 records unique to WoSCC, 168 unique to Scopus, and 325 shared by both databases. For duplicate records, the version containing the most complete bibliographic information and abstract was retained, together with database-source indicators. The complete matching rules and results are provided in the Supplementary Material.

Keyword co-occurrence analyses were performed separately within the WoSCC and Scopus datasets. At the document level, keywords were standardized for capitalization, spacing, and hyphenation. Singular and plural forms, spelling variants, abbreviations and their full forms, and semantically equivalent terms were merged. Demographic indexing terms, generic reporting terms, and noise terms lacking thematic discriminative value were removed. Because keyword fields and indexing mechanisms differ between the two databases, database-specific networks were constructed and interpreted separately, without directly comparing unstandardized raw frequencies. The merged and deduplicated dataset of 541 publications was used exclusively for LDA topic modeling and was not used to generate a merged keyword co-occurrence network. The keyword-cleaning thesaurus, term-merging rules, and frequency thresholds are provided in the Supplementary Material.

### LDA topic modeling and trend analysis

English-language abstracts from the merged and deduplicated WoSCC and Scopus dataset were used to construct the latent Dirichlet allocation (LDA) corpus. Text preprocessing included text standardization, removal of stop words and noise terms, lemmatization, and standardization of domain-specific multiword expressions. Publications without an abstract or with an insufficient number of valid tokens after preprocessing were excluded from modeling. A total of 524 publications were ultimately included in the primary LDA analysis.

The final five-topic model was selected through an integrated evaluation of model fit, topic stability, model parsimony, and domain interpretability across candidate topic numbers. Topics were interpreted and labeled according to their highest-probability terms, relevance-ranked terms, and representative publications. Topic labels were researcher-generated summaries of the statistical topics and did not contribute to model estimation. The complete corpus-construction procedure, vocabulary-screening criteria, model-selection metrics, hyperparameters, random seed, iteration settings, and stability assessment results are provided in the Supplementary Material.

To explore temporal changes in research themes, the annual mean posterior probability of each topic was calculated for 2015–2025, and its relationship with publication year was examined. Regression slopes, 95% confidence intervals, two-sided P values, and coefficients of determination (R²) were reported, with the Benjamini–Hochberg procedure applied to adjust for multiple comparisons. Given the limited number of annual observations and the compositional nature of topic probabilities, these findings were interpreted as exploratory.

To assess the sensitivity of the topic structure to database source, models were additionally fitted to the WoSCC-only, Scopus-only, and merged deduplicated corpora using the same text-preprocessing procedure and the same number of topics (K = 5). Single-database topics were compared with the merged model in terms of topic-term composition and the direction of annual changes. Candidate topic-number comparisons, model parameters, repeated runs, bootstrap stability assessments, and database-source sensitivity analyses are reported in Supplementary Tables S9–S17 and Supplementary Figures S1–S2.

## Results

### Publication output, geographic distribution, and collaboration patterns

**Fig. 2:**
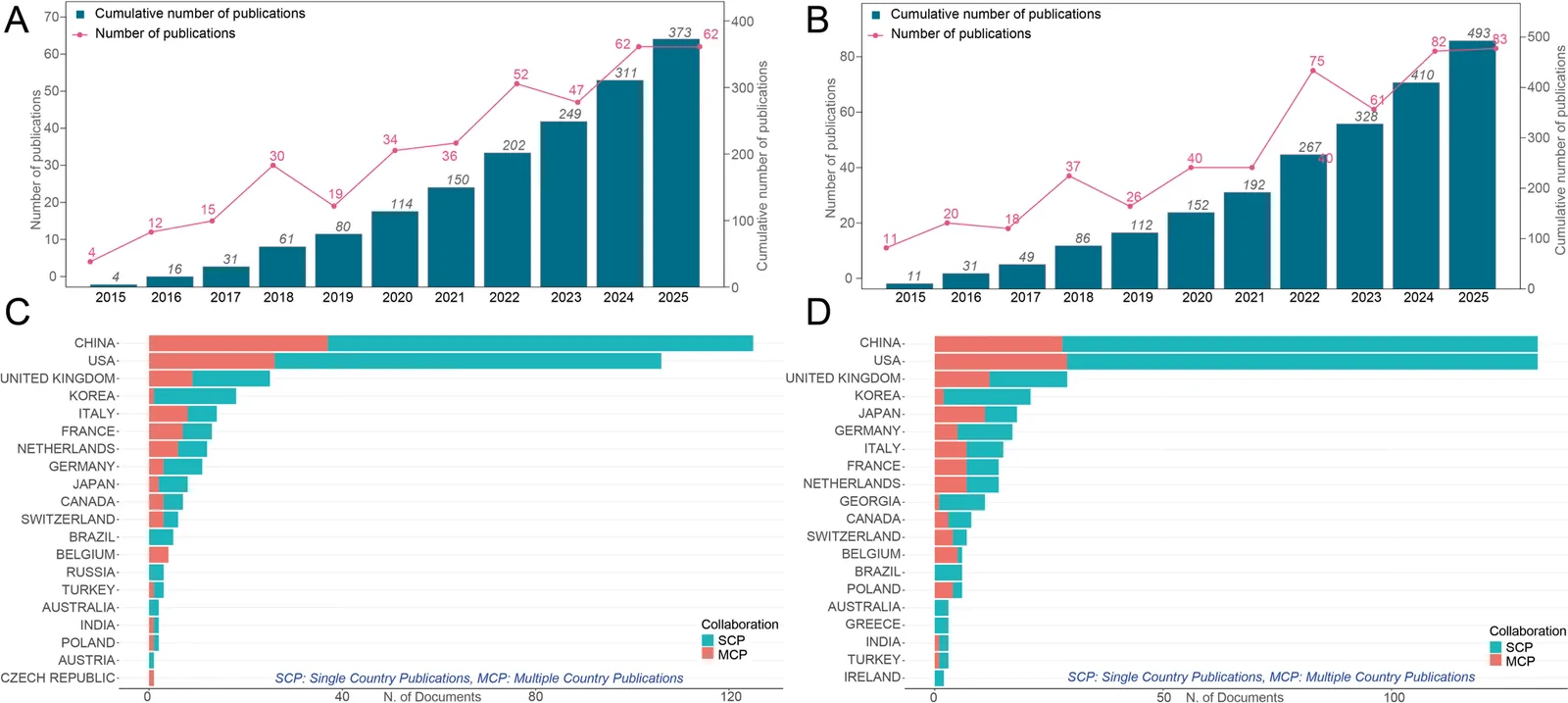
(**A**) Annual and cumulative publications in WoSCC; (**B**) Annual and cumulative publications in Scopus; (**C**) Distribution of publications by corresponding-author country in WoSCC; (**D**) Distribution of publications by corresponding-author country in Scopus

From 2015 to 2025, 373 publications from WoSCC and 493 publications from Scopus were included. Annual publication output in WoSCC increased from 4 publications in 2015 to 62 in 2025, whereas that in Scopus increased from 11 to 83 publications. Despite fluctuations in certain years, both databases exhibited an overall upward trend in annual publication output, which remained at a relatively high level during the latter part of the study period (Fig. 2A, B). As shown in Fig. 2C and D, China ranked first and the United States second in WoSCC, whereas China and the United States each contributed 132 publications (26.8%) in Scopus and jointly ranked first. China contributed 104 single-country publications and 28 multiple-country publications, while the corresponding numbers for the United States were 103 and 29, respectively. Their MCP proportions were 21.2% and 22.0%, respectively. The United Kingdom ranked immediately behind these two countries in both databases, while South Korea, Japan, Germany, Italy, France, and the Netherlands also demonstrated substantial research activity.

Table 1 further identifies China and the United States as the principal contributing countries, with the United Kingdom, Japan, Germany, Italy, and the Netherlands forming a second tier of research contributors. The distribution of SCPs and MCPs indicates that research output from China and the United States consisted predominantly of single-country publications, although both countries also participated in substantial international collaboration. Publications from South Korea were produced primarily by domestic research teams. By comparison, Japan, Canada, Switzerland, Italy, the Netherlands, Germany, France, and the United Kingdom exhibited more pronounced multinational collaboration patterns, indicating greater participation in international research collaborations.

**Table 1:** Top 10 corresponding-author countries by publication count and characteristics of international collaboration in WoSCC and Scopus

Country	Articles	Freq (%)	SCP	MCP	MCP Ratio (%)
**WoSCC**					
CHINA	137	25.9	87	50	36.5
USA	120	22.7	81	39	32.5
UNITED KINGDOM	36	6.8	16	20	55.6
JAPAN	32	6	6	26	81.3
GERMANY	22	4.2	8	14	63.6
NETHERLANDS	21	4	7	14	66.7
ITALY	21	4	6	15	71.4
SOUTH KOREA	18	3.4	17	1	5.6
CANADA	17	3.2	4	13	76.5
SWITZERLAND	17	3.2	3	14	82.4
**Scopus**					
USA	173	25.8	127	46	26.6
CHINA	147	21.9	102	45	30.6
UNITED KINGDOM	44	6.6	18	26	59.1
JAPAN	38	5.7	7	31	81.6
GERMANY	32	4.8	13	19	59.4
ITALY	27	4	8	19	70.4
NETHERLANDS	23	3.4	10	13	56.5
SOUTH KOREA	21	3.1	19	2	9.5
FRANCE	19	2.8	8	11	57.9
CANADA	19	2.8	5	14	73.7

**Fig. 3:**
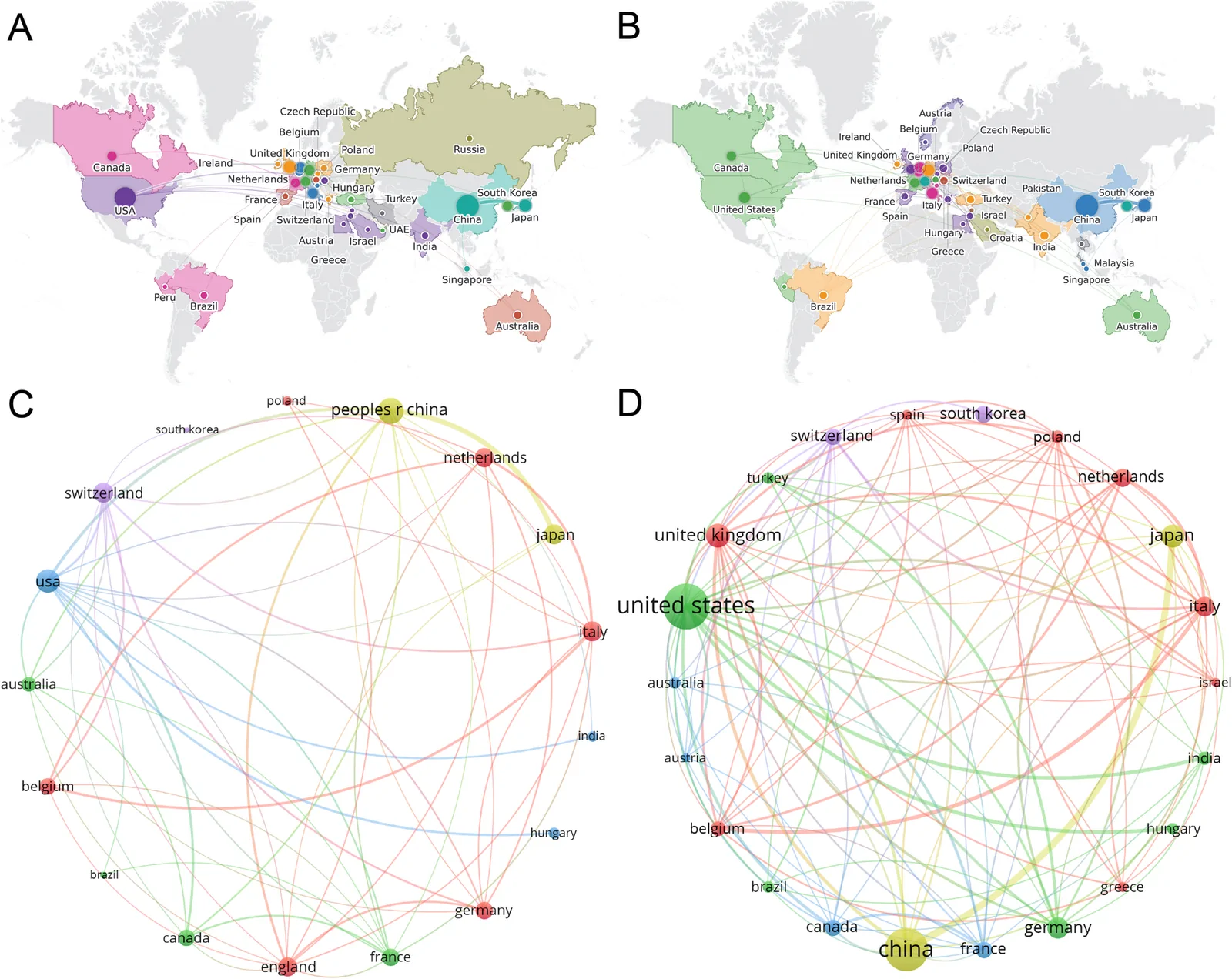
(**A**) Geographical distribution of publications in WoSCC; (**B**) geographical distribution of publications in Scopus; (**C**) country collaboration network in WoSCC; (**D**) country collaboration network in Scopus

The geographical distributions shown in Fig. 3A and B indicate that RVIS research was concentrated primarily in East Asia, North America, and Western Europe, while also extending to Oceania, South America, and other regions. Both WoSCC and Scopus identified China and the United States as major national research hubs in this field, with the United Kingdom, Germany, Italy, France, the Netherlands, Switzerland, Japan, and South Korea forming active regional research networks. The country collaboration networks further revealed an international collaborative structure spanning Asia, North America, and Europe. China and the United States occupied central positions in these networks. The United States maintained extensive collaborative links with multiple European and Asian countries, whereas China collaborated with major research contributors across Asia, Europe, and North America. Collaboration among European countries was particularly dense, with the United Kingdom, Italy, Germany, France, the Netherlands, and Switzerland serving as important connecting nodes among different country clusters. Japan also represented an important node within the Asian collaboration network (Fig. 3C, D).

Based on fractional counting of institutional contributions, Beijing Institute of Technology ranked among the leading institutions in both databases. Imperial College London, the University of California San Diego, Kagawa University, Hanyang University, Houston Methodist Hospital, and the University of Twente also demonstrated relatively high research output in both datasets. In addition, Shanghai Jiao Tong University, the Chinese Academy of Sciences, and the Chinese University of Hong Kong were prominent in WoSCC, whereas the Georgia Institute of Technology, the Shenzhen Institutes of Advanced Technology, and the Frederik Meijer Heart & Vascular Institute were among the most productive institutions in Scopus (Table 2).

**Table 2:** Top 10 research institutions by publication count in WoSCC and Scopus

Affiliation	Fractionalized articles
**WoSCC**	
Beijing Institute of Technology	12.69
Imperial College London	10.87
University of California, San Diego	10.37
Shanghai Jiao Tong University	6.37
Kagawa University	6.28
Hanyang University	5.98
Houston Methodist Hospital	5.25
Chinese University of Hong Kong	4.94
Chinese Academy of Sciences	4.72
University of Twente	4.63
**Scopus**	
Beijing Institute of Technology	16.07
University of California, San Diego	12.03
Kagawa University	9.48
Georgia Institute of Technology	7.83
Houston Methodist Hospital	7.62
Imperial College London	7.03
Hanyang University	6.58
University of Twente	5.99
Shenzhen Institutes of Advanced Technology	5.98
Frederik Meijer Heart & Vascular Institute	5.65

### Journal and co-cited journal analysis

**Fig. 4:**
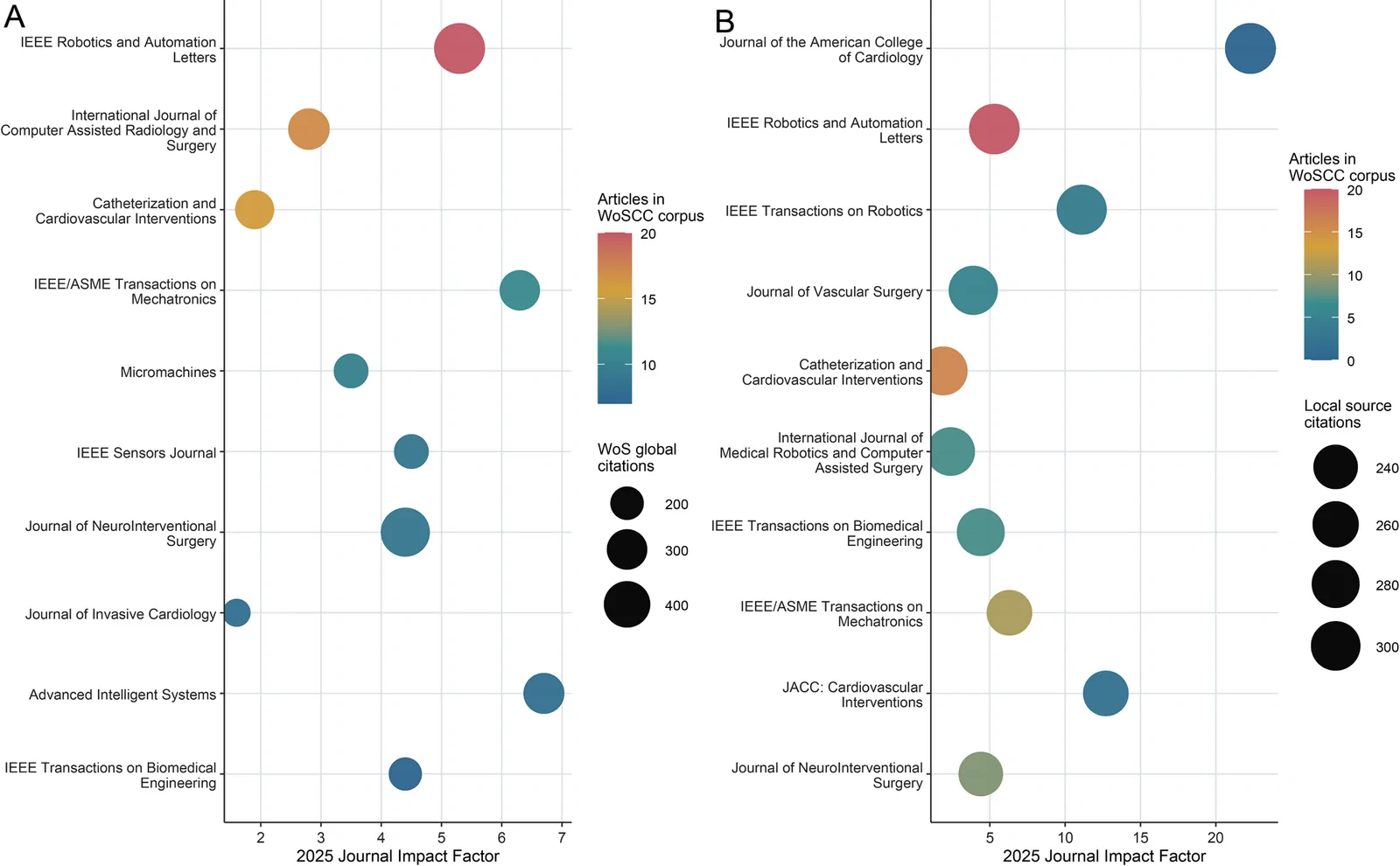
(**A**) Top 10 most productive sources; (**B**) top 10 most locally cited sources

The journal distributions in WoSCC and Scopus showed substantial overlap. IEEE Robotics and Automation Letters, International Journal of Computer Assisted Radiology and Surgery, and Catheterization and Cardiovascular Interventions were among the leading source journals in both databases. In addition, WoSCC included a relatively large number of publications from IEEE/ASME Transactions on Mechatronics, Micromachines, Journal of NeuroInterventional Surgery, and IEEE Transactions on Biomedical Engineering. Scopus provided broader coverage of publications from IEEE Transactions on Medical Robotics and Bionics, The International Journal of Medical Robotics and Computer Assisted Surgery, Cardiovascular Revascularization Medicine, and Biomedical Microdevices. Overall, research in this field was primarily published in journals covering robotics, mechatronics, biomedical engineering, computer-assisted surgery, and cardiovascular and neurovascular interventions, demonstrating a distinct interdisciplinary interface between engineering and clinical medicine (Fig. 4A; Table 3).

**Table 3:** Top 10 source journals by publication count in WoSCC and Scopus

Journal	Articles	Citations	IF (2025)	Journal	Articles	IF (2025)
WoSCC				Scopus		
IEEE Robotics and Automation Letters	20	484	5.3	IEEE Robotics and Automation Letters	19	5.3
International Journal of Computer Assisted Radiology and Surgery	17	316	2.8	International Journal of Computer Assisted Radiology and Surgery	19	2.8
Catheterization and Cardiovascular Interventions	16	278	1.9	Catheterization and Cardiovascular Interventions	17	1.9
IEEE/ASME Transactions on Mechatronics	11	299	6.3	IEEE Transactions on Medical Robotics and Bionics	14	4.3
Micromachines	10	217	3.5	Micromachines	12	3.5
Journal of NeuroInterventional Surgery	9	448	4.4	Journal of NeuroInterventional Surgery	10	4.4
IEEE Sensors Journal	9	217	4.5	IEEE/ASME Transactions on Mechatronics	10	6.3
Advanced Intelligent Systems	8	307	6.7	International Journal of Medical Robotics and Computer Assisted Surgery	10	2.4
Journal of Invasive Cardiology	8	136	1.6	Cardiovascular Revascularization Medicine	9	2.3
IEEE Transactions on Biomedical Engineering	7	197	4.4	Biomedical Microdevices	8	3.3

IF (2025) denotes the 2025 Journal Impact Factor reported in the 2026 edition of Clarivate Journal Citation Reports (JCR), accessed on September 3, 2026

Local citation analysis within the WoSCC dataset showed that IEEE Robotics and Automation Letters, Journal of the American College of Cardiology, IEEE Transactions on Robotics, Journal of Vascular Surgery, and Catheterization and Cardiovascular Interventions were important citation sources for the included literature. IEEE Transactions on Biomedical Engineering, JACC, Cardiovascular Interventions, and Journal of NeuroInterventional Surgery also received relatively high local citation counts. These journals encompass robotic system design, biomedical engineering, vascular surgery, and cardiovascular and neurovascular interventions, indicating that the intellectual base of the field draws on both engineering research and clinical interventional studies (Fig. 4B; Table 4).

**Table 4:** Top 10 source journals by local citation count in WoSCC

Rank	Journal	Cites	Articles	IF (2025)
1	IEEE Robotics and Automation Letters	323	20	5.3
2	Journal of the American College of Cardiology	317	0	22.3
3	IEEE Transactions on Robotics	307	4	11.1
4	Journal of Vascular Surgery	295	5	3.9
5	Catheterization and Cardiovascular Interventions	293	16	1.9
6	International Journal of Medical Robotics and Computer Assisted Surgery	288	7	2.4
7	IEEE Transactions on Biomedical Engineering	279	7	4.4
8	JACC: Cardiovascular Interventions	255	2	12.7
9	IEEE/ASME Transactions on Mechatronics	252	11	6.3
10	Journal of NeuroInterventional Surgery	240	9	4.4

IF (2025) denotes the 2025 Journal Impact Factor reported in the 2026 edition of Clarivate Journal Citation Reports (JCR), accessed on September 3, 2026

**Fig. 5:**
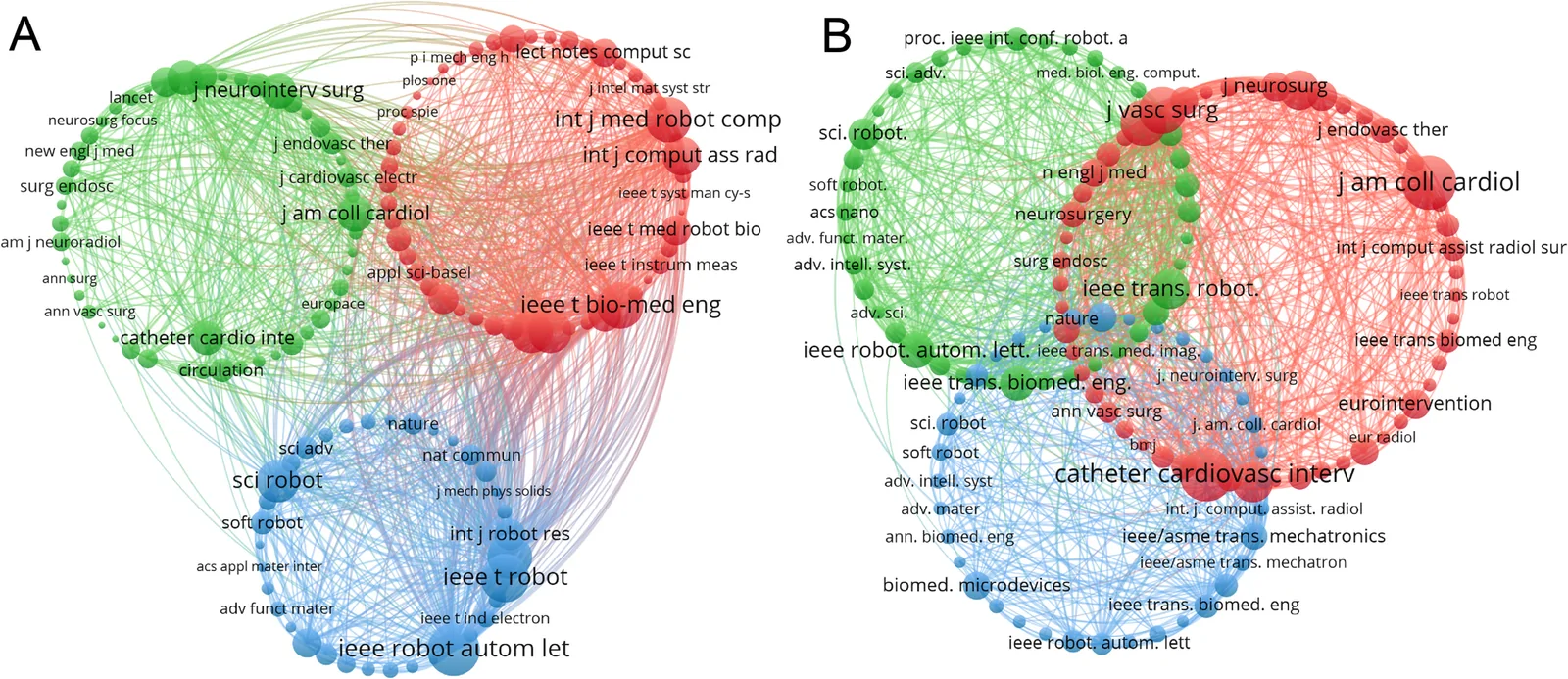
(**A**) Co-citation network of cited sources in WoSCC; (**B**) corresponding network in Scopus

The WoSCC journal co-citation network comprised three interconnected knowledge clusters. The first, represented by IEEE Transactions on Biomedical Engineering, The International Journal of Medical Robotics and Computer Assisted Surgery, and International Journal of Computer Assisted Radiology and Surgery, primarily reflected research on medical robotics, biomedical engineering, and computer-assisted surgery. The second, represented by Journal of the American College of Cardiology, Catheterization and Cardiovascular Interventions, Journal of Vascular Surgery, and Journal of NeuroInterventional Surgery, encompassed cardiovascular medicine, vascular surgery, and neurointerventional research. The third, represented by IEEE Robotics and Automation Letters, IEEE Transactions on Robotics, and IEEE/ASME Transactions on Mechatronics, mainly involved robotic control, mechatronics, and related engineering technologies (Fig. 5A). The Scopus journal co-citation network exhibited a comparable interdisciplinary structure. Robotics and biomedical engineering journals, computer-assisted surgery journals, and clinical journals related to cardiovascular and neurovascular interventions jointly formed the principal knowledge clusters, with extensive co-citation links among them (Fig. 5B). Networks from both databases indicated that the intellectual base of RVIS spans robotics and control technologies, biomedical engineering, and clinical interventional medicine. However, the sizes and network positions of individual journal nodes differed because of variations in database coverage and reference-indexing practices.

### Author contributions and collaboration networks

The fractional-counting analysis showed that Guo S had the highest fractional publication count and fractional citation count in both WoSCC and Scopus. Lumsden AB, Riga C, Mahmud E, and Desai JP also ranked among the leading authors by fractional publication output in both databases. In addition, Britz GW, Lu Q, and Bismuth J demonstrated sustained research contributions across both datasets, whereas Li Y and Omisore OM had particularly prominent fractional contributions in Scopus (Table 5).

**Table 5:** Top 10 most productive authors in WoSCC and Scopus

Authors	Fractionalized documents	Fractionalized citations
**WoSCC**		
Guo, Shuxiang	5.58	163.54
Lumsden, Alan B.	3.1	61.44
Riga, Celia	2.27	59.99
Mahmud, Ehtisham	2.16	77.35
Desai, Jaydev P.	2.08	53.42
Britz, Gavin W.	1.89	43.54
Tamiya, Takashi	1.64	62.48
Bismuth, Jean	1.63	22.55
Wang, Lei	1.51	59.21
Lu, Qingsheng	1.46	30.79
**Scopus**		
Guo, Shuxiang	5.53	279.15
Lumsden, Alan B	4.35	80.39
Riga, Celia	2.74	88.46
Mahmud, Ehtisham	2.65	103.29
Desai, Jaydev P.	2.55	52.47
Britz, Gavin W.	2.39	53.26
Lu, Qingsheng	2.11	61.95
Li, Youxiang	1.83	107.36
Omisore, Olatunji Mumini	1.82	69.33
Bismuth, Jean	1.8	27.66

**Fig. 6:**
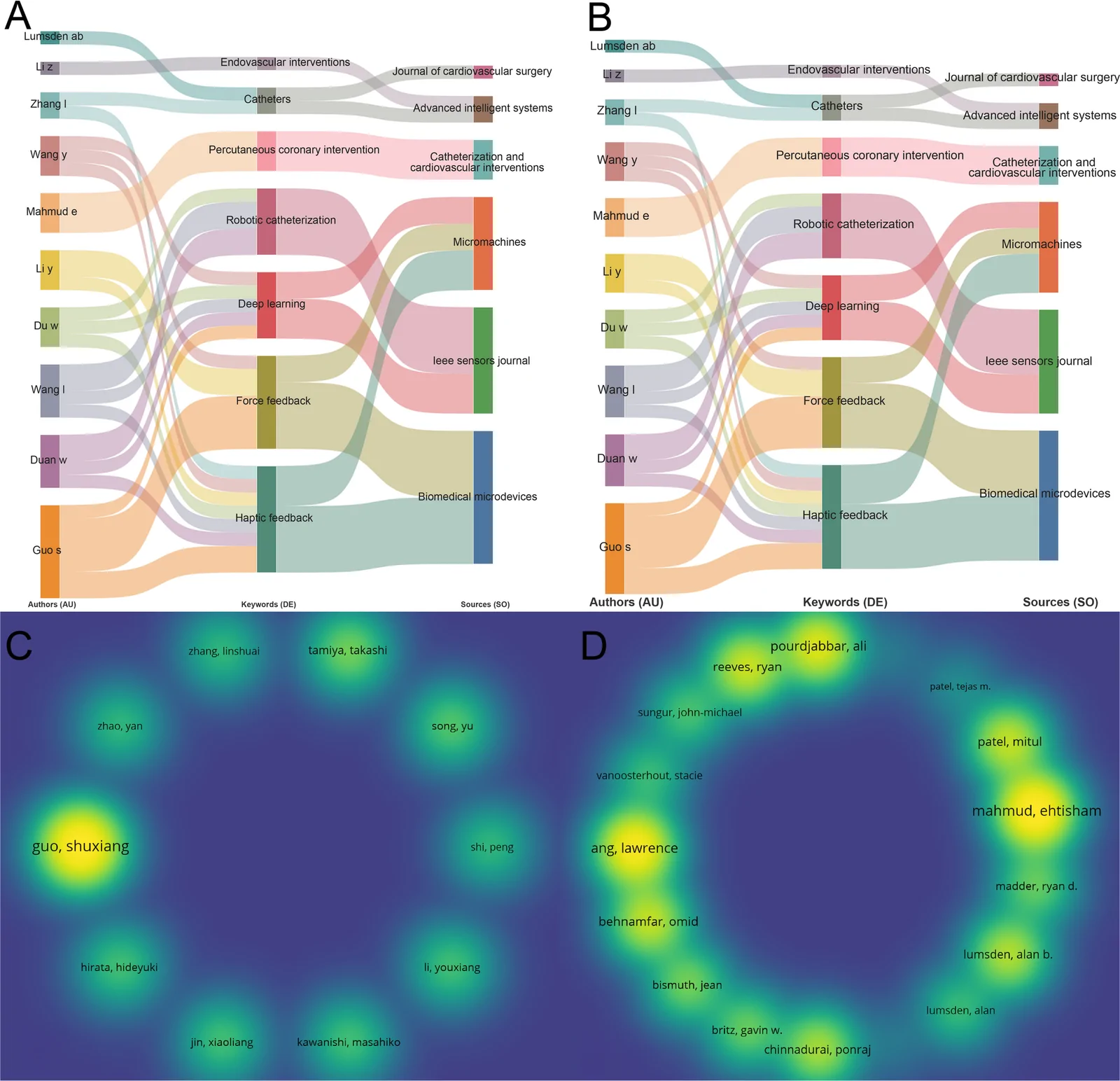
(**A**) Three-field plot of authors, author keywords, and source journals in WoSCC; (**B**) three-field plot of authors, index keywords, and source journals in Scopus; (**C**) co-authorship subnetwork in WoSCC; (**D**) co-authorship network in Scopus

The three-field plot for WoSCC showed that Guo S was primarily associated with technical topics such as force feedback, haptic feedback, and deep learning, with the corresponding publications appearing mainly in Biomedical Microdevices and Micromachines. Mahmud E was predominantly associated with percutaneous coronary intervention, with related studies published in Catheterization and Cardiovascular Interventions. Lumsden AB, Zhang L, and Li Z were more closely connected to topics such as catheters and endovascular interventions (Fig. 6A). The Scopus three-field plot similarly revealed a parallel structure of engineering research and clinical intervention. Authors including Duan W, Du W, and Wang L were primarily associated with robotic catheterization, deep learning, and haptic feedback, and their corresponding publications were concentrated in IEEE Sensors Journal, Micromachines, and Biomedical Microdevices (Fig. 6B).

In the WoSCC author collaboration density map, Guo S formed the most prominent high-density area, while several secondary areas of activity were observed around Tamiya T, Hirata H, Song Y, and Kawanishi M. The Scopus network displayed multiple, relatively dispersed collaboration hotspots. Areas involving Mahmud E, Ang L, Pourdjabbar A, and Reeves R were particularly active, while Lumsden AB, Britz GW, Patel M, and Madder RD also formed identifiable collaborative groups. Together, the two networks showed that author collaboration in this field comprised both engineering teams focusing on robotic systems, control, and feedback technologies and clinical teams focusing on coronary and endovascular interventions. However, collaboration between these different research groups remained relatively limited (Fig. 6C, D).

The fractional citation analysis in WoSCC showed that Guo S had the highest fractionalized total citation count, followed by Zhao X, Wang L, Guo CF, and Kim Y. After adjustment for citation accumulation time using fractionalized citations per year, Wang L, Guo S, and Zhao X remained among the leading authors, indicating that their publications received sustained citation attention within the included dataset. Mahmud E, Zhang L, Harker P, Patel AB, and Tamiya T also demonstrated relatively high fractional citation performance (Table 6).

**Table 6:** Top 10 authors by cumulative citations in WoSCC

Rank	Authors	Fractionalized citations	Fractionalized TC per year	Documents
1	Guo, Shuxiang	163.54	26.75	30
2	Zhao, Xuanhe	153.45	26.22	4
3	Wang, Liu	142.31	27.81	5
4	Guo, Chuan Fei	116.25	18.78	3
5	Kim, Yoonho	95.39	15.8	3
6	Zhang, Li	81.09	19.82	9
7	Mahmud, Ehtisham	77.35	8	11
8	Harker, Pablo	72.37	13.3	2
9	Patel, Aman B.	72.37	13.3	2
10	Tamiya, Takashi	62.48	8.27	9

### Highly cited publications and citation burst analysis

The highly cited publications in WoSCC primarily addressed magnetically actuated guidewires and soft robots, robot-assisted endovascular catheter manipulation, intelligent navigation, and robot-assisted percutaneous coronary intervention. The study by Kim et al. on magnetically controlled remote neurovascular intervention ranked among the leading publications in both total and annualized citation counts. Jeon et al. investigated guidewire actuation using a magnetically controlled soft microrobot, while Omisore et al. focused on vascular interventional robotic systems based on intelligent control. Although the study by Dreyfus et al. on intravascular navigation using a helical magnetic robot was published relatively recently, it demonstrated a high annualized citation rate. The clinical study by Mahmud et al. on robot-assisted intervention for complex coronary lesions was also among the highly cited publications (Table 7).

**Table 7:** Top 10 globally cited documents in WoSCC

Paper	DOI	Total Citations	TC per Year
KIM, 2022, SCI ROBOT	https://doi.org/10.1126/SCIROBOTICS.ABG9907	372	74.4
JEON, 2019, SOFT ROBOT	https://doi.org/10.1089/SORO.2018.0019	260	32.5
WANG, 2021, P NATL ACAD SCI USA	https://doi.org/10.1073/PNAS.2021922118	211	35.17
OMISORE, 2022, IEEE T SYST MAN CY-S	https://doi.org/10.1109/TSMC.2020.3026174	202	40.4
DREYFUS, 2024, SCI ROBOT	https://doi.org/10.1126/SCIROBOTICS.ADH0298	152	50.67
HU, 2018, COMPUT ASSIST SURG	https://doi.org/10.1080/24699322.2018.1526972	151	16.78
MAHMUD, 2017, JACC-CARDIOVASC INTE	https://doi.org/10.1016/J.JCIN.2017.03.050	151	15.1
PATEL, 2019, ECLINICALMEDICINE	https://doi.org/10.1016/J.ECLINM.2019.07.017	139	17.38
YANG, 2023, ADV INTELL SYST-GER	https://doi.org/10.1002/AISY.202200416	139	34.75
WANG, 2022, NAT COMMUN	https://doi.org/10.1038/S41467-022-32059-9	133	26.6

The highly cited publications identified in Scopus similarly encompassed magnetically actuated robots, robotic catheter control, neurovascular interventions, and robot-assisted coronary interventions. Studies by Kim, Jeon, Omisore, and Mahmud et al. received substantial citation attention in both databases. In addition, the study by Guo et al. on a robot-assisted endovascular catheter system with haptic feedback, the study by Patel et al. on remote robotic coronary intervention, and the study by Pereira et al. on robot-assisted neurovascular intervention ranked among the most highly cited publications in Scopus. Collectively, the findings from both databases indicate that frequently cited research included not only engineering advances in robotic structures, actuation, and control but also clinical feasibility studies of coronary and neurovascular interventions (Table 8).

**Table 8:** Top 10 globally cited documents in Scopus

Paper	DOI	Total Citations	TC per Year
KIM, 2022, Science Robotics	https://doi.org/10.1126/SCIROBOTICS.ABG9907	381	76.2
JEON, 2019, Soft Robotics	https://doi.org/10.1089/SORO.2018.0019	275	34.38
OMISORE, 2022, Ieee Transactions On Systems	https://doi.org/10.1109/TSMC.2020.3026174	230	46
GUO, 2019, Ieee Transactions On Robotics	https://doi.org/10.1109/TRO.2019.2896763	173	21.63
MAHMUD, 2017, Jacc: Cardiovascular Interventions	https://doi.org/10.1016/J.JCIN.2017.03.050	173	17.3
PATEL, 2019, Eclinicalmedicine	https://doi.org/10.1016/J.ECLINM.2019.07.017	161	20.13
PEREIRA, 2020, Journal Of Neurointerventional Surgery	https://doi.org/10.1136/NEURINTSURG-2019-015671.REP	149	21.29
YANG, 2023, Advanced Intelligent Systems	https://doi.org/10.1002/AISY.202200416	143	35.75
DREYFUS, 2024, Science Robotics	https://doi.org/10.1126/SCIROBOTICS.ADH0298	137	45.67
WANG, 2022, Nature Communications	https://doi.org/10.1038/S41467-022-32059-9	133	26.6

**Fig. 7:**
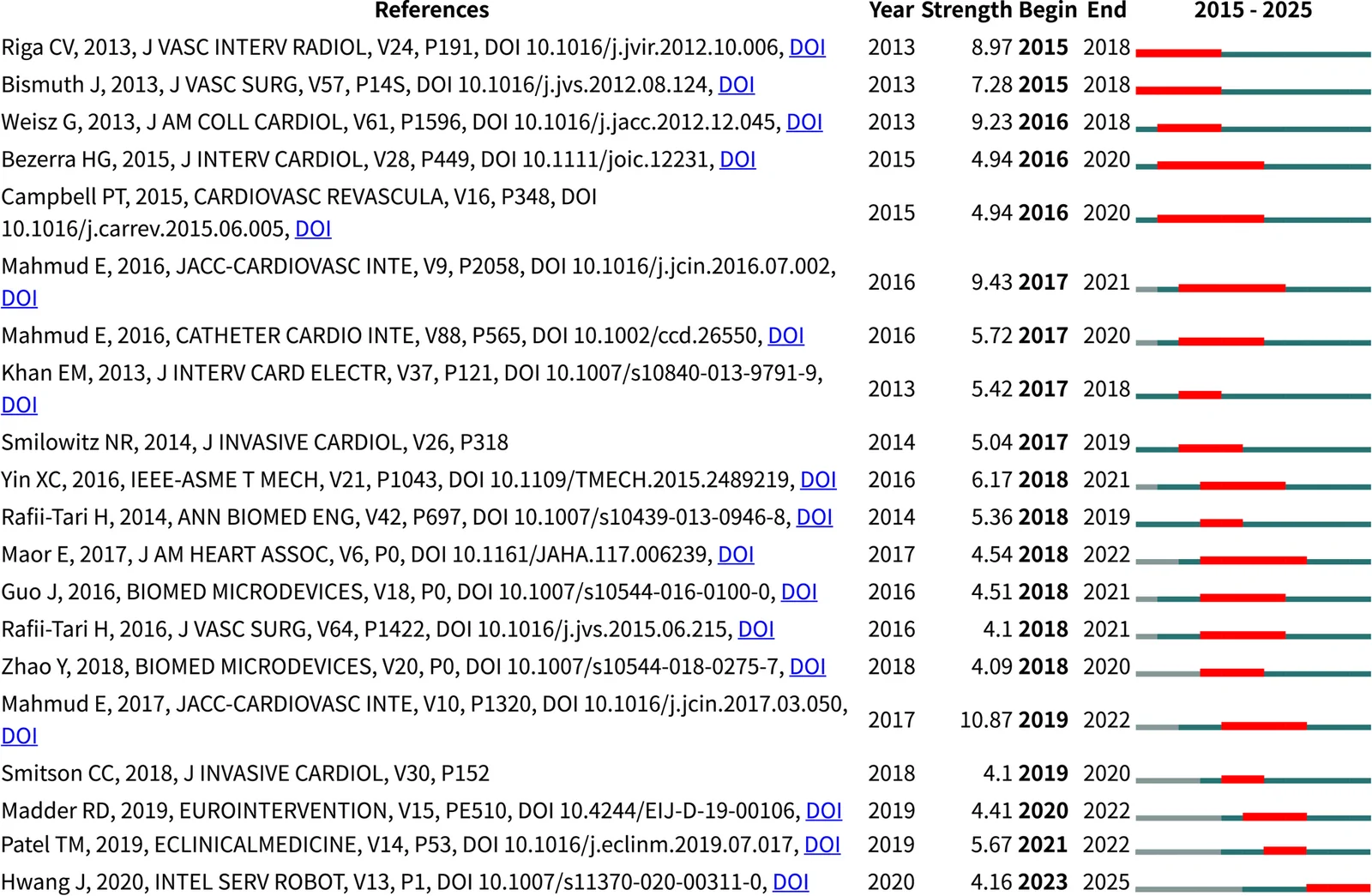
The 20 most strongly burst-cited references in WoSCC

The citation-burst analysis shown in Fig. 7 revealed temporal changes in the studies receiving concentrated citation attention within the field. From 2015 to 2018, early studies by Riga, Bismuth, and Weisz et al. on robot-assisted endovascular procedures and coronary interventions were among the first to exhibit citation bursts. After 2016, research attention further extended to radiation protection, safety, and the clinical feasibility of robot-assisted percutaneous coronary intervention. The CORA-PCI study published by Mahmud et al. in 2017 exhibited the strongest citation burst (burst strength = 10.87), which continued through 2022, indicating that robot-assisted intervention for complex coronary lesions received substantial citation attention during this period. Meanwhile, engineering studies by Yin, Rafii-Tari, Guo, and Zhao et al. on robotic system design, catheter manipulation, force feedback, and intravascular navigation also successively exhibited citation bursts. Toward the end of the study period, the review by Hwang et al. on magnetically actuated systems and guidewire- and catheter-based microrobots showed a sustained citation burst from 2023 to 2025, indicating that magnetically actuated vascular interventional robots have attracted concentrated citation attention in recent years.

### Keyword co-occurrence and topic evolution

Table 9 showed that both WoSCC and Scopus covered core topics such as robotics, endovascular, robot-assisted surgery, and percutaneous coronary intervention. However, their frequencies and rankings were affected by differences in database coverage and keyword fields. Publications in WoSCC more frequently addressed catheters, navigation, and force feedback, whereas Scopus provided broader coverage of clinical applications such as coronary artery disease, robot-assisted PCI, and vascular intervention. The findings from the two databases were used to describe shared research interests and database-specific differences rather than as a formal assessment of robustness.

**Table 9:** Frequency rankings of author keywords in WoSCC and Scopus

Keyword	Count	Keyword	Count
WoSCC author keywords		Scopus author keywords	
Robotics	85	Robotics	91
Endovascular	37	Percutaneous coronary intervention	59
Robot-assisted surgery	36	Robot-assisted surgery	41
Percutaneous coronary intervention	29	Endovascular	27
Catheter	24	Coronary Artery Disease	23
Endovascular intervention	17	Endovascular intervention	20
Medical Robotics	15	Medical Robotics	18
Navigation	15	Robotic percutaneous coronary intervention	15
Intervention	14	Endovascular Robotics	13
Force Feedback	11	Vascular Interventional Surgery	13

**Fig. 8:**
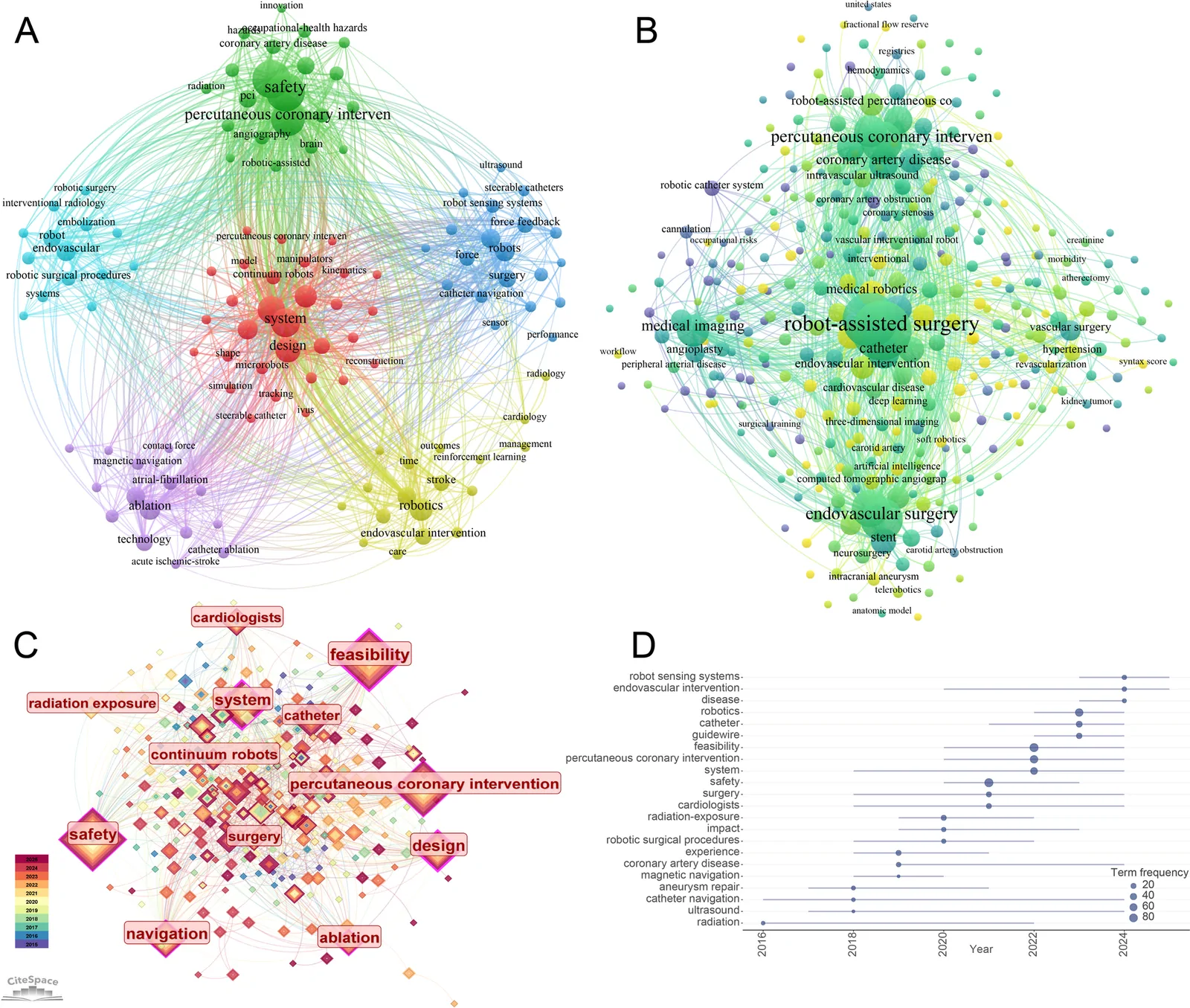
(**A**) All keyword co-occurrence network in WoSCC; (**B**) indexed keyword co-occurrence network in Scopus; (**C**) temporal keyword network in WoSCC; (**D**) thematic trends in WoSCC

The WoSCC keyword co-occurrence network comprised six interconnected research clusters (Fig. 8A). The cluster centered on system, navigation, design, catheter, and guidewire mainly encompassed robotic system design, catheter and guidewire manipulation, and navigation technologies. The cluster characterized by safety, feasibility, and percutaneous coronary intervention focused on clinical feasibility, procedural safety, and applications in coronary intervention. Another cluster, represented by robots, catheter, force feedback, and robot sensing systems, reflected research on robotic catheter systems, sensing, and force feedback. The remaining clusters addressed robotics, radiation exposure, endovascular intervention and stroke; catheter ablation, haptic feedback, and magnetic navigation; and endovascular treatment, embolization, and interventional imaging.

The Scopus keyword co-occurrence network similarly encompassed both engineering technologies and clinical applications (Fig. 8B). At the engineering level, robot-assisted surgery, robotics, catheter, medical robotics, and endovascular intervention formed a major cluster that included robotic catheter systems, magnetic navigation, remote manipulation, force feedback, and haptic feedback. At the clinical level, percutaneous coronary intervention, fluoroscopy, radiation exposure, coronary angiography, and safety formed a cluster related to coronary intervention and radiation protection. Other interrelated clinical research directions were represented by keywords concerning thrombectomy, stent implantation, therapeutic embolization, interventional imaging, angioplasty, and endovascular aortic repair. Networks from both databases demonstrated extensive connections among robotic system development, sensing and navigation technologies, and vascular interventional applications.

The temporal evolution of WoSCC keywords showed that earlier publications more frequently addressed radiation, ultrasonography, and aneurysm repair. Research subsequently expanded to include procedural experience, system performance, safety, feasibility, and percutaneous coronary intervention. In recent years, keywords such as guidewire, robots, endovascular intervention, robotics, and catheter remained highly active, reflecting an increasing concentration of research attention on guidewire and catheter manipulation, robotic system design, and endovascular applications. Overall, the keyword structure expanded from an early emphasis on the procedural environment and clinical problems to the evaluation of system feasibility and key technologies for robotic intervention, while maintaining continuity among themes across different periods (Fig. 8C, D).

### LDA topic identification and evolutionary trends

The LDA model identified five principal topics, Topic 1, catheter teleoperation and haptic feedback; Topic 2, remote image-guided platforms; Topic 3, magnetically actuated soft robots and microrobots; Topic 4, robot-assisted PCI and clinical outcomes; and Topic 5, vascular surgery and thrombectomy. Based on the mean posterior topic probabilities, Topic 2 had the highest overall prevalence (0.3012), followed by Topic 4 (0.2302), Topic 3 (0.1789), Topic 1 (0.1702), and Topic 5 (0.1194). When publications were assigned to their dominant topics according to the maximum posterior probability, Topic 2 also contained the largest number of publications, indicating that teleoperation, interventional imaging, device tracking, and radiation protection constituted a broadly represented research direction within the field (Supplementary Table S11).

**Fig. 9:**
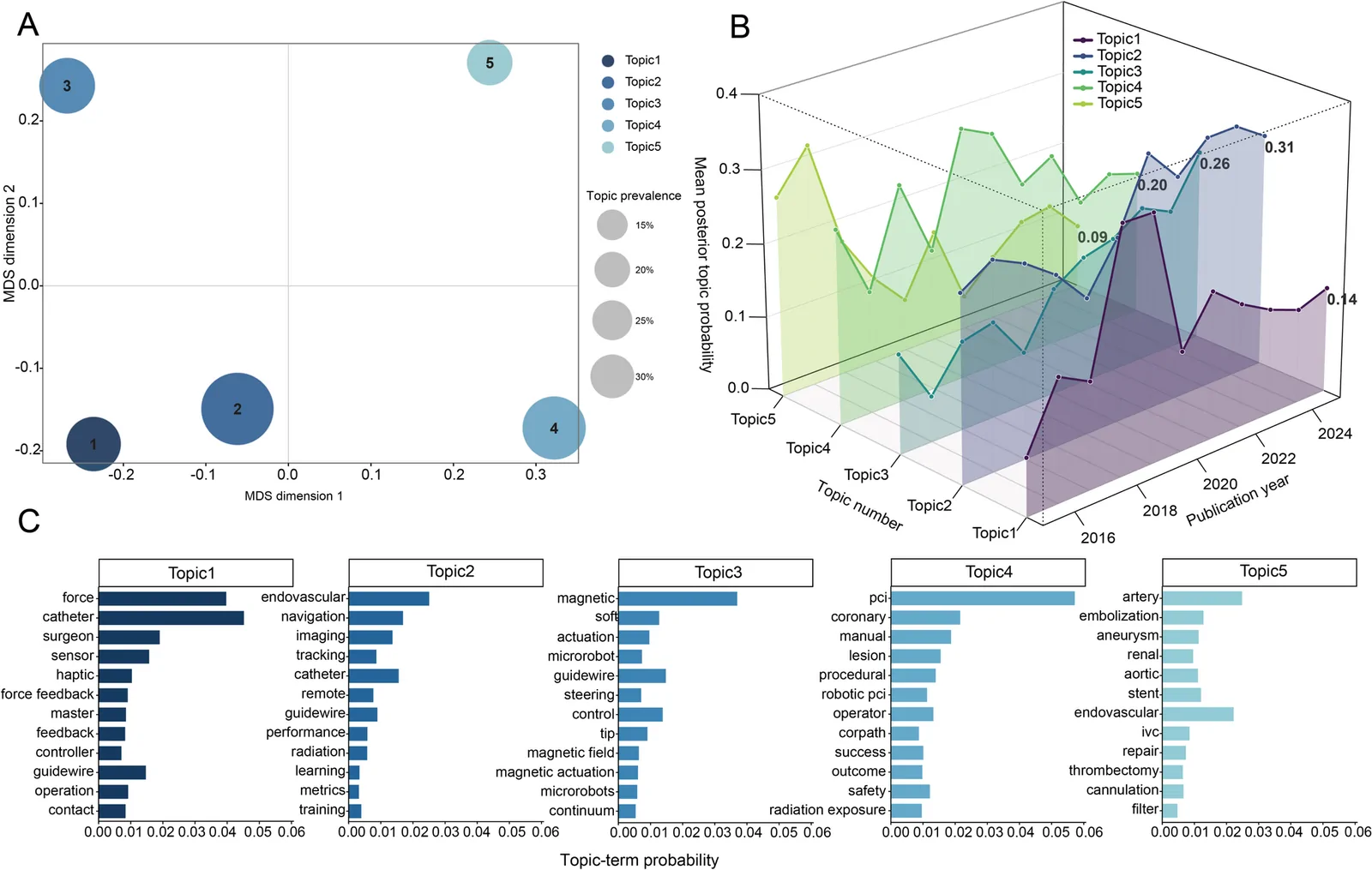
LDA topic modeling results based on the merged deduplicated corpus. (**A**) Intertopic distance map; (**B**) temporal trends of the five topics from 2015 to 2025; (**C**) representative terms of the five topics

The representative terms further characterized the specific content of each topic. Topic 1 was primarily defined by catheter, force, sensor, haptic feedback, guidewire, and control, reflecting catheter and guidewire teleoperation, force sensing, haptic feedback, and controller design. Topic 2 was characterized by endovascular, navigation, imaging, tracking, remote, and radiation, encompassing remote image guidance, device tracking, and radiation protection. Topic 3 included magnetic, soft, actuation, microrobot, steering, and guidewire, representing magnetically actuated soft robots, microrobots, and intravascular steering technologies. Topic 4 centered on PCI, coronary, lesion, robotic PCI, operator, and procedural outcome, with an emphasis on procedural performance, safety, and clinical outcomes in robot-assisted coronary intervention. Topic 5 was principally associated with artery, embolization, aneurysm, aortic, thrombectomy, and IVC, covering aneurysm treatment, embolization, thrombectomy, and other vascular surgical applications (Fig. 9C).

The intertopic distance map showed varying degrees of relative separation among the five topics in the two-dimensional space, indicating differences in their term distributions. However, the multidimensional scaling axes have no specific clinical or disciplinary meaning; therefore, the spatial distribution was not used to define distinct disciplinary boundaries (Fig. 9A). Exploratory temporal trend analysis of the merged corpus showed a significant increase in the relative prevalence of Topic 3, magnetically actuated soft robots and microrobots (slope = 0.01474 per year, 95% CI: 0.00743–0.02205, *P* = 0.00136, BH-adjusted *P* = 0.00680, R² = 0.6982). Topic 5, vascular surgery and thrombectomy, showed a decreasing trend in the merged corpus (slope = − 0.01795 per year, 95% CI: −0.03211 to − 0.00379, *P* = 0.01854, BH-adjusted *P* = 0.04635, R² = 0.4775). Topic 2 showed an increasing trend before adjustment but did not remain statistically significant after Benjamini–Hochberg correction. No significant linear trends were identified for Topic 1 or Topic 4 (Supplementary Table S12; Fig. 9B).

The database-source sensitivity analysis yielded mean matched-topic Jaccard similarities of 0.546 for the WoSCC-only corpus and 0.684 for the Scopus-only corpus relative to the merged model, indicating a moderate degree of reproducibility for the principal thematic structure. Topic 3 showed the same upward direction across all three corpora. By contrast, the downward direction of Topic 5 was not reproduced in the WoSCC-only corpus and was therefore interpreted only as an exploratory finding from the merged corpus (Supplementary Tables S15–S17; Supplementary Figures. S1–S2).

## Discussion

### From peripheral exploration to broader research attention

The bibliometric analysis showed an overall increase in RVIS-related publications from 2015 to 2025, with publication output remaining relatively high in 2024 and 2025. This pattern indicates increasing research activity and scholarly attention in the field. However, the annual publication trajectory alone cannot demonstrate that the technology has crossed a critical threshold of innovation diffusion or entered an accelerated phase of clinical translation. Previous clinical and technical studies provide contextual explanations for this growth. The CorPath GRX system received CE marking for neurovascular interventions in 2018, and the first-in-human robot-assisted neurovascular intervention was performed in 2019 [14]. Meanwhile, advances in deep learning for medical image processing have facilitated research on vision-based force feedback, autonomous navigation, and the integration of multimodal imaging with robotic platforms [17, 18]. These external developments may have coincided with the increase in research output; however, causal relationships between them cannot be established from publication counts. Therefore, the higher-output years are more appropriately interpreted as periods of concentrated publication activity rather than direct boundaries between distinct stages of technological or clinical development.

Bibliometric data do not contain information on hospital adoption rates, actual procedural volumes, or clinical penetration. Consequently, this study cannot directly compare the growth of scientific publications with the pace of clinical adoption. As external context, previous literature has reported that more than 13 million robot-assisted procedures performed worldwide have predominantly involved the da Vinci system in urology, gynecology, and general surgery, whereas the use of robotic technologies in vascular surgery and vascular interventions remains comparatively limited [19]. Although systems such as CorPath GRX and R-One have received regulatory approval and accumulated some clinical evidence, previous reviews continue to identify equipment costs, training systems, regulatory requirements, workflow integration, and insufficient long-term clinical evidence as barriers to wider implementation [15, 20, 21]. An increase in publication output therefore indicates growing scholarly attention, whereas assessments of the extent of clinical implementation and its associated barriers should be based on the cited clinical studies and technical reviews rather than directly quantified from the publication trajectory observed in this study.

### A reflection of interdisciplinary convergence

At the country level, China and the United States ranked among the top two contributors in both databases and represented the principal national research forces in this field, although their relative rankings differed. China ranked first and the United States second in WoSCC, whereas the two countries jointly ranked first in Scopus. This geographical distribution is broadly consistent with the core regions of North America, Western Europe, and East Asia identified in previous robotic surgery research [20]. In the broader field of vascular surgery, China has also exhibited marked publication growth in artificial intelligence and digital health research [21]. Differences in country rankings between the databases may be attributable to variations in journal coverage, disciplinary scope, and indexing practices. The two datasets should therefore be regarded as qualitatively complementary representations of the research landscape rather than as a formal demonstration of robustness. Because this study did not compare journal tiers or mean citation impact at the country level, no conclusions can be drawn regarding the proportion of publications from individual countries appearing in high-impact journals or their average scholarly influence.

At the institutional level, both engineering institutions and clinical centers were represented among the most productive organizations. Previous systematic mapping of institutions involved in the development of robotic platforms has similarly identified collaboration between engineering universities and clinical centers [1], while reviews of remote vascular intervention robots have further emphasized the importance of interdisciplinary teamwork [22]. This institutional composition indicates that RVIS research depends on collaboration between engineering technology development, the definition of clinical needs, and application-based validation.

Fractional author contributions and collaboration networks revealed several research groups with distinct areas of emphasis. Collaboration was relatively concentrated within the principal research teams, whereas connections among different teams remained limited, suggesting scope for further cross-institutional and interdisciplinary collaboration. It should be noted that even under fractional counting, author rankings may remain sensitive to database coverage, team size, and a small number of highly cited publications. These findings therefore represent authors’ relative contributions and the citation attention received within the corpus analyzed in this study and should not be interpreted as direct measures of their overall scholarly influence.

### Evolution of research hotspots and intelligent research directions

Keyword co-occurrence and temporal evolution analyses showed that the scope of RVIS research expanded over different periods. Earlier publications more frequently addressed radiation, ultrasonography, and aneurysm repair. Safety, feasibility, system performance, and percutaneous coronary intervention subsequently received sustained attention, while keywords such as guidewire, catheter, robots, and endovascular intervention remained highly active in recent years. These changes suggest that research attention has expanded from the procedural environment and clinical problems to key technologies for robot-assisted intervention. However, they do not establish a unidirectional or inevitable transition among research themes.

Advances in embodied intelligence, autonomous navigation, and deep reinforcement learning provide an external technological context for these keyword changes. Conventional master–slave systems depend primarily on real-time manipulation by the operator, whereas embodied intelligence research seeks to enable robots to integrate imaging, sensing, and information acquired through interaction with the physical environment to support path planning and procedural assistance [17, 18]. Relevant reviews have discussed potential technological pathways from teleoperation toward supervised autonomy [23], including the use of long short-term memory networks to model catheter hysteresis and the training of guidewire-navigation strategies in simulated or animal vascular environments [1]. These studies indicate that environmental perception and decision support have become active areas of engineering research. Nevertheless, the bibliometric findings reported here reflect only the level of research attention and do not demonstrate that autonomous interventional systems have reached technological maturity or routine clinical application.

The LDA results further characterized changes in the thematic structure of research at the semantic level. Among the five identified topics, the relative prevalence of magnetically actuated soft robots and microrobots remained significantly increased after correction for multiple comparisons, indicating that this research direction received growing relative attention during the study period. Rotationally actuated continuum robots have been shown to address the limitations of conventional guidewire advancement by converting rotation into forward propulsion through contact between a helical surface and the vessel wall, thereby enabling navigation from the aortic arch into millimeter-scale cerebral arteries [23]. In the merged corpus, the relative prevalence of the vascular surgery and thrombectomy topic decreased. However, this direction was not reproduced in the WoSCC-only model, indicating sensitivity to database composition. It should therefore not be interpreted as evidence of an absolute decline in the number or importance of such clinical studies.

### Clinical translation and implementation challenges

The discussion in this section regarding technological feasibility, clinical adoption, costs, regulation, and implementation barriers is based primarily on previous clinical studies and technical reviews. It is intended to provide contextual interpretation for the bibliometric patterns observed in this study and does not represent information directly measured by the bibliometric dataset. Relevant publications from 2026 were not included in the 2015–2025 analytical corpus and are cited only as supplementary technological and clinical context beyond the predefined study period.

Despite increasing research activity in RVIS, widespread clinical adoption continues to face multidimensional challenges. In published clinical studies, robot-assisted neurovascular intervention achieved a technical success rate of approximately 93%, while the rate of unplanned conversion to manual operation remained 7%, primarily because of anatomical difficulty, device malfunction, and limitations in catheter length [24]. From a broader perspective, five major challenges have been systematically identified: equipment costs of approximately US$650,000 per system, absence of haptic feedback, limited capacity for coordinated manipulation of multiple devices, lack of standardized training systems, and insufficient evidence regarding long-term clinical outcomes [25]. From a clinical perspective, realizing the objective of intelligent vascular robotic surgery will require further advances in operator trust, intuitive operation, and fault tolerance during emergencies [26].

The journal co-citation networks contained interconnected clusters of engineering and clinical interventional journals, reflecting the interdisciplinary intellectual base of RVIS. After Benjamini–Hochberg correction for multiple comparisons, the relative topic weight of Topic 3, magnetically actuated soft robots and microrobots, showed an increasing trend, whereas Topic 1, catheter teleoperation and haptic feedback, showed no clear linear change. Accordingly, the following discussion of haptic feedback and clinical implementation is based primarily on previous technical and clinical literature rather than on an increasing trend directly identified by the LDA analysis.

Evidence from previous technical and clinical studies indicates that inadequate haptic feedback remains an important challenge for RVIS. During conventional manual procedures, interventionalists perceive the contact force between the guidewire and the vessel wall through their fingertips, which assists in determining device position and reducing the risk of vascular injury. By contrast, existing robotic systems continue to rely predominantly on visual feedback. Visual force feedback, vibrotactile feedback, and other force-sensing approaches have been investigated experimentally, but their feedback accuracy, signal latency, stability, and synchronization with imaging systems require further optimization and validation [10–12]. Moreover, when equipment failure or an acute vascular event occurs, the operator may need to undock the robotic system and convert to manual operation. The time required for this process and its potential effect on emergency management, particularly in neurovascular interventions with a narrow margin for error, should be evaluated in future clinical studies and incorporated into standardized emergency protocols [24, 25].

From a health-economic perspective, high capital costs of approximately US$650,000 per system, together with recurring expenditure on consumables, limit the accessibility of robotic platforms in many medical centers. Comparative studies of robot-assisted and manual PCI have shown that robotic assistance can substantially reduce operator radiation exposure, with a mean reduction of 95.2%. However, procedural duration has not been consistently shortened, and the incremental cost-effectiveness of the technology remains uncertain [27]. Regarding clinical effectiveness, prospective nonrandomized trials have reported mid-term follow-up outcomes after robot-assisted cerebral aneurysm embolization, supporting the feasibility of robot-assisted neurovascular intervention [28]. Other comparative studies have found no significant differences in major adverse cardiovascular events between robotic and manual procedures, although robotic assistance may offer advantages in the precision of stent deployment [29]. Current evidence therefore remains insufficient to determine whether the incremental cost of robot-assisted procedures produces a corresponding incremental clinical benefit.

In addition to equipment and consumable costs, the safe implementation of RVIS depends on institutional maintenance capacity, procedural volume, staffing, and team training. Previous reviews have indicated that high acquisition and maintenance costs, the procedural volume required to maintain economic viability, and limited access to robotic training may restrict adoption across medical institutions and contribute to a learning curve [25]. Institutions implementing RVIS therefore require not only trained interventionalists but also standardized procedures involving interventional, anesthesia, nursing, and engineering personnel for equipment preparation, malfunction management, rapid undocking, and conversion to manual operation [25, 26]. Because direct comparative evidence on regional differences in RVIS complication rates remains unavailable, this study does not interpret resource disparities as established regional differences in procedural safety. Instead, these disparities are considered potential factors that may affect implementation consistency and emergency-response capacity. Future studies should use standardized training, simulation exercises, credentialing systems, and multicenter clinical registries to evaluate relationships between institutional resources and safety outcomes.

Beyond device performance and costs, the safe implementation of robot-assisted intervention also involves anesthetic management, ventilation monitoring, staffing, and emergency response. Literature on anesthesia for robotic surgery indicates that, after robotic docking, equipment configuration may restrict the anesthesia team’s access to the patient, airway, and vascular lines. Endotracheal tubes, monitoring lines, and infusion lines should therefore be secured before docking, and an emergency undocking protocol should be established in advance [30]. During prolonged procedures requiring general anesthesia and mechanical ventilation, bedside intervention may be delayed if endotracheal tube displacement, secretion obstruction, or sudden changes in respiratory mechanics occur. Studies of ventilation during robotic surgery have emphasized continuous monitoring of oxygenation, end-tidal carbon dioxide, tidal volume, and airway pressure, together with individualized lung-protective ventilation strategies based on the patient’s respiratory mechanics [31].

Importantly, the available evidence on ventilation is derived primarily from robot-assisted urological and gynecological surgery. Pneumoperitoneum and specialized patient positioning in these procedures are not inherent features of RVIS, and their associated pulmonary risks should therefore not be directly extrapolated to RVIS. Dedicated clinical studies are required to determine whether robotic docking, the fluoroscopic environment, and prolonged procedural duration increase delays in airway intervention or ventilation-related risks during RVIS. Furthermore, as robotic systems expand toward remote operation, intelligent navigation, and decision support, regulatory evaluation will need to address software updates, network and data security, system-failure management, and the capacity for human takeover [32]. When device malfunction, communication failure, or algorithmic output contributes to patient harm, the allocation of responsibility among operators, healthcare institutions, and device manufacturers may become increasingly complex. No consensus has yet been established regarding liability for highly autonomous surgical robotic systems [33].

These challenges have not prevented continued exploration of advanced technologies. Recent studies have begun to investigate the application of embodied intelligence in vascular interventional robots [23]. A central limitation of conventional master–slave architectures is that robots passively execute commands without autonomous perception. By contrast, embodied intelligence emphasizes learning and adaptation through continuous interaction with the physical environment. This transition is being driven by the convergence of deep learning, computer vision, and reinforcement learning. Real-time vessel segmentation, device tracking, and anatomical landmark detection provide robotic systems with the capacity to perceive vascular anatomy, while deep Q-networks and imitation learning enable them to learn and reproduce expert manipulation strategies [34].

Several technological directions have undergone exploratory investigation and preliminary validation. In autonomous navigation, the hierarchical autonomous guidewire navigation and delivery framework proposed in 2026 integrated multimodal large language models with hierarchical reinforcement learning and reported improved guidewire localization and targeted delivery in complex vascular environments [34]. A deep learning-based human–robot collaborative navigation framework used a generative adversarial network to achieve real-time local path planning for the catheter tip, with a reported action-decision accuracy of 93.75%.

Regarding novel robotic platforms, the SENTANTE vascular interventional robotic system has undergone a first-in-human trial. The system enables fully remote manipulation of standard guidewires, sheaths, balloons, stents, coils, and other interventional devices; can simultaneously manipulate up to three telescoping devices; and provides real-time force feedback intended to approximate the experience of bedside manipulation [35]. A portable haptic robotic device weighing only 400 g achieved millimeter-level positioning accuracy and autonomous guidewire advancement using servo-current sensing and impedance control. In stenosis models, it achieved F-scores ranging from 0.83 to 0.95 for lesion-stiffness classification [36]. For coordinated manipulation of multiple devices, a high-precision force-sensing system based on lever amplification shortened the force-transmission pathway and enhanced sensitivity to subtle vessel–device interaction forces [37]. In remote intervention, a vascular interventional control system based on WebRTC and 5G communication demonstrated feasibility and safety in a remote clinical trial [38]. At the clinical validation level, the randomized NAVIGATE GRX study showed that the TechnIQ automation algorithms integrated into the CorPath GRX system shortened robot-assisted PCI procedural time without increasing the rate of unplanned manual conversion [39].

Collectively, these advances suggest that RVIS research is exploring a potential evolution from passive tools directly controlled by the operator toward intelligent collaborators with environmental perception and decision-support capabilities [23]. Under this framework, the operator may progressively shift from direct manipulation toward higher-level supervision, while the robotic system assists with precise device manipulation, path planning, and risk identification. Whether these research directions can be translated into safe, effective, and accessible clinical systems will depend substantially on cost control, standardized validation, and the parallel development of appropriate regulatory frameworks.

## Limitations

This study has several limitations. First, only publications in English were included, potentially excluding relevant studies published in other languages and introducing language bias. Second, citation indicators are subject to time-dependent accumulation. Publications appearing toward the end of the study period may therefore have been underestimated because of their shorter citation windows. Although annualized citation indicators were also reported, they cannot completely eliminate the influence of publication year. Third, bibliometric data primarily reflect publication output, citation relationships, and changes in research attention. They cannot directly measure the technological maturity of robotic systems, hospital adoption rates, actual procedural volumes, cost-effectiveness, or patient outcomes. Bibliometric findings therefore cannot substitute for clinical trials, real-world studies, or health-economic evaluations. Fourth, WoSCC and Scopus differ inherently in database coverage, disciplinary scope, and keyword-indexing mechanisms. Although cross-database comparisons were restricted to author keywords generated in a comparable manner and terminology was standardized, differences in the completeness of author-keyword fields and database-specific indexing practices may still have affected keyword frequencies and network structures. The findings from the two databases should therefore be regarded as descriptive comparisons rather than a formal demonstration of robustness. Fifth, network analysis may be affected by minimum occurrence frequencies, node-inclusion thresholds, counting methods, normalization approaches, and network-pruning parameters. Although these settings improve the readability of network visualizations, they may also result in the underrepresentation of low-frequency nodes or weaker relationships. Sixth, LDA-derived topics are sensitive to text preprocessing, vocabulary-selection thresholds, the number of candidate topics, and random initialization. Although different candidate topic numbers and parameter combinations were evaluated and bootstrap stability assessments were performed, overall topic stability remained moderate. Furthermore, topic labeling required researchers to interpret high-weight terms and representative publications. Alternative reasonable preprocessing procedures or model specifications may therefore produce different thematic structures. Seventh, the temporal trend analysis was based on only 11 annual observations from 2015 to 2025 and was affected by the compositional nature of topic probabilities, unequal publication counts across years, and potential nonlinearity and temporal correlation. Although the Benjamini–Hochberg procedure was applied to adjust for multiple testing, this correction cannot eliminate the limitations arising from the data structure and small number of time points. The corresponding findings should therefore be interpreted only as exploratory evidence of changes in relative research attention. Eighth, author-name disambiguation and institutional-name standardization were based primarily on names, affiliations, coauthorship relationships, and available ORCID information. A small number of authors with identical names may not have been fully distinguished, and some name variants may not have been completely consolidated. Finally, self-citations were not excluded separately; author and journal self-citations may therefore have influenced citation counts and the corresponding rankings to some extent.

## Conclusion

Overall, publication output in RVIS increased between 2015 and 2025. The field encompassed mechanical design and master–slave control, human–robot interaction, force sensing and haptic feedback, image-guided navigation, magnetically actuated systems, and intelligent decision support, forming an intellectual structure that connects engineering technologies with clinical interventional medicine. The topic of magnetically actuated soft robots and microrobots showed an upward direction across all three corpora, whereas the boundaries and temporal trajectories of the other topics demonstrated some sensitivity to database source. The bibliometric and LDA findings reflect scholarly output, citation relationships, and relative research attention. They do not establish that the technology has completed a transition between developmental stages and cannot be used to directly infer clinical adoption, technological maturity, or patient benefit. Future research should further evaluate procedural safety across different vascular anatomical settings, quantify the relationships between force-feedback accuracy, control latency, and clinical outcomes, and validate the safety, effectiveness, generalizability, and scalability of image-guided navigation, force sensing, motion control, and machine learning-assisted decision-support systems through standardized multicenter studies.

## Supplementary Information

Below is the link to the electronic supplementary material.

Supplementary Material 1

## Data Availability

All data analyzed in this study were retrieved from the Web of Science Core Collection and Scopus databases, both of which are publicly accessible repositories. The complete search strategies, inclusion and exclusion criteria, and detailed parameter settings for VOSviewer, CiteSpace, and Bibliometrix are provided in the Supplementary Material accompanying this article. No primary experimental data were generated.
