# Supplementary material for: Bibliometric and knowledge-map analysis of research on robot-assisted vascular interventional surgery (2015–2025)
Source: J Robot Surg. 2026 Sep 12;20(1):983. doi: 10.1007/s11701-026-03947-9 (PMC13570900; doi:10.1007/s11701-026-03947-9)
Supplement: Supplementary file 1 [file 11701_2026_3947_MOESM1_ESM.docx]

**Supplementary Material**

# 1. Database searches and staged record selection

**Web of Science Core Collection (Science Citation Index Expanded)**

Search strategy: TS=((robot* OR "robotic-assisted" OR "robot-assisted" OR "interventional robot") AND ("vascular intervention" OR "endovascular" OR "percutaneous coronary" OR "vascular surgery" OR "intravascular" OR "endovascular intervention")).

Search execution date: July 1, 2026.

The original export contained 690 records. Restriction to articles and reviews retained 609 records; restriction to English retained 606; restriction to 2015–2025 retained 592; and removal of one retracted publication retained 591 records for title/abstract eligibility screening. Application of the final operational definition excluded 218 topic-ineligible records, yielding 373 WoS records.

**Scopus**

Search strategy: TITLE-ABS-KEY((robot* OR "robotic-assisted" OR "robot-assisted" OR "interventional robot") AND ("vascular intervention" OR "endovascular" OR "percutaneous coronary" OR "vascular surgery" OR "intravascular") AND ("surgery" OR "intervention" OR "procedure" OR "treatment")).

Search execution date: July 1, 2026.

The search yielded 1,616 records. Restriction to articles and reviews, English language, and publication years 2015–2025 retained 1,061 records. Application of the same final operational definition excluded 567 topic-ineligible records, leaving 494 eligible records. One exact internal duplicate was then removed, yielding 493 Scopus records.

**Supplementary Table S1. Sequential screening flow for each database.**

| **Database** | **Screening stage** | **Records retained** | **Records removed at stage** |
| --- | --- | --- | --- |
| WoS | Initial export | 690 | — |
| WoS | Article/Review | 609 | 81 |
| WoS | English | 606 | 3 |
| WoS | 2015–2025 | 592 | 14 |
| WoS | Non-retracted | 591 | 1 |
| WoS | Topic eligible | 373 | 218 |
| Scopus | Initial retrieval | 1,616 | — |
| Scopus | Article/Review, English, 2015–2025 | 1,061 | 555 |
| Scopus | Topic eligible | 494 | 567 |
| Scopus | Internal deduplication | 493 | 1 |

# 2. Unified operational definition and eligibility boundaries

Robot-assisted vascular interventional surgery was operationally defined as a catheter-based, guidewire-based, or actively navigated intravascular/endovascular procedure in which a robotic system directly assists or controls vascular instrument manipulation, navigation, positioning, or therapeutic delivery.

Inclusion criteria

•	Robot-assisted percutaneous coronary intervention, neurovascular intervention, peripheral vascular intervention, and other catheter-based vascular procedures.

•	Robotic or telerobotic catheter/guidewire manipulation, steering, navigation, positioning, force sensing, haptic feedback, or autonomous control developed for vascular intervention.

•	Robot-assisted transcatheter structural-heart or electrophysiology procedures when the robotic system directly manipulated or navigated the intravascular catheter.

•	Active magnetic micro-/milli-robots, magnetic guidewires, or magnetic catheters used for controlled intravascular navigation, recanalization, embolization, thrombectomy, localized delivery, or related vascular therapy.

•	Clinical, preclinical, engineering, and review articles whose central focus met the operational definition.

Exclusion criteria

•	Open, laparoscopic, or thoracoscopic robotic vascular surgery, vascular anastomosis, aneurysm resection/repair, robotic CABG, and robot-assisted bypass surgery without robotic intravascular manipulation.

•	Hybrid coronary revascularization when the robot was used only for surgical grafting and the PCI component was manual; hybrid reports were included only when robotic PCI was explicitly performed.

•	Vascular injuries or complications arising from unrelated robotic oncologic, urologic, thoracic, orthopedic, or gastrointestinal surgery.

•	Non-robotic endovascular treatment, generic vascular imaging, AI segmentation, clinical outcome comparisons, or simulation studies without a central robotic intervention system.

•	Passive/general DNA nanorobot or nanomedicine drug-delivery studies without active controlled operation in the vascular lumen.

•	Non-intravascular procedures such as robotic radiosurgery, focused ultrasound treatment, and retinal or microsurgical procedures that did not use catheter-/guidewire-based vascular navigation.

**Supplementary Table S2. Prespecified decisions for recurrent boundary cases.**

| **Boundary case** | **Decision** | **Rationale** |
| --- | --- | --- |
| Robotic PCI / robotic coronary angiography | Include | Robot directly manipulates intravascular coronary instruments. |
| Robotic carotid, peripheral, renal-denervation, or IVC-filter intervention | Include | Catheter-/guidewire-based vascular intervention. |
| Magnetic micro-/milli-robot in vessels | Include | Active intravascular navigation or therapy; passive nanomedicine excluded. |
| Robot-assisted transcatheter structural-heart intervention | Include | Included only when the robot directly manipulates the catheter. |
| Robotic CABG or robotic MIDCAB | Exclude | Robot performs surgical grafting rather than intravascular intervention. |
| Hybrid CABG + PCI | Conditional | Include only if the PCI component is explicitly robot assisted. |
| Da Vinci vascular aneurysm repair/resection | Exclude | Open/laparoscopic vascular surgery outside the operational definition. |
| AI imaging or vessel segmentation | Exclude | No central robotic catheter/guidewire system. |
| Robotic radiosurgery for AVM | Exclude | Not an intravascular procedure. |
| Generic DNA nanorobot drug delivery | Exclude | No controlled intravascular robotic navigation/manipulation. |

# 3. Database-specific yield and conceptual comparability

The two databases were screened using the same document-type, language, publication-year, and content-based eligibility criteria. Eligibility was determined by study content rather than by a target database size.

For WoS, 591 records remained after metadata eligibility screening and removal of one retracted publication. Content-based screening excluded 218 records outside the operational definition and retained 373 records.

For Scopus, 1,061 records remained after the combined document-type, language, and year restrictions. Content-based screening excluded 567 records, and removal of one exact internal duplicate retained 493 records. The larger Scopus yield reflects broader source coverage and the use of title, abstract, and keyword fields; database totals were not made similar through selective exclusion.

Because database coverage, indexing practices, and citation structures differ, database-specific descriptive, citation, and network results were analyzed separately. Similar patterns across databases were treated as qualitative complementarity rather than as a formal robustness test.

# 4. Topic-exclusion audit

Each excluded record was assigned one primary hierarchical reason. Counts below therefore sum to the topic-exclusion totals without double counting. The complete title-level audit is supplied separately as Supplementary Data S1.

**Supplementary Table S3. Primary topic-exclusion reasons after metadata screening.**

| **Code** | **Primary exclusion reason** | **WoS** | **Scopus** |
| --- | --- | --- | --- |
| E01 | No robotic system or robotics not central to the study | 1 | 4 |
| E02 | No intravascular/endovascular intervention context | 13 | 82 |
| E03 | Indirect/non-central robot–vascular linkage; generic AI, imaging, outcomes, or non-robotic enabling study | 136 | 269 |
| E04 | Open/laparoscopic/thoracoscopic robotic surgery, CABG, bypass, or surgical vascular repair | 63 | 204 |
| E05 | Vascular complication of unrelated robotic surgery | 4 | 8 |
| E06 | Passive/general nanomedicine without active intravascular robotics | 1 | 0 |
| Total | Topic exclusions | 218 | 567 |

# 5. Internal and cross-database deduplication

Within each database, DOI was normalized by removing URL prefixes, converting to lower case, and trimming terminal punctuation. When DOI was missing, normalized title, publication year, journal, and authors were compared. One exact Scopus duplicate was removed; the DOI-containing version was retained.

Across databases, 317 record pairs were matched by exact normalized DOI and eight additional pairs by exact normalized title, giving 325 exact cross-database pairs. For each pair, the WoS record was retained as the primary merged record. Forty-eight WoS records and 168 Scopus records remained unmatched. Approximate title screening used publication year (±1 year), character similarity (≥0.86), and token Jaccard similarity (≥0.78); no additional fuzzy candidate met these thresholds. The merged corpus therefore comprised 541 unique publications.

Merged flow: 373 WoS + 168 Scopus-unique = 541 publications

**Supplementary Table S4. Cross-database consolidation results.**

| **Item** | **Count** |
| --- | --- |
| Final WoS records | 373 |
| Final Scopus records after internal deduplication | 493 |
| Pairs matched by normalized DOI | 317 |
| Additional pairs matched by normalized title | 8 |
| All exact cross-database pairs | 325 |
| Unmatched WoS records | 48 |
| Unmatched Scopus records | 168 |
| Additional fuzzy duplicate candidates | 0 |
| Merged deduplicated corpus | 541 |

# 6. Author, institution, and country normalization

Author names were standardized for punctuation, spacing, repeated initials, and compatible missing middle names. Full names, affiliations, co-authorship patterns, and ORCID identifiers were cross-referenced where available. Ambiguous cases were not automatically merged. Institution abbreviations and full-name variants were merged only when they denoted the same organization; country variants were mapped to a canonical country name.

**Supplementary Table S5. Representative normalization examples.**

| **Original variation** | **Canonical form** |
| --- | --- |
| Lumsden, Alan / Lumsden, Alan B. | Lumsden, Alan B |
| Riga, Celia / Riga, Celia V. | Riga, Celia V |
| Britz, Gavin / Britz, Gavin W. | Britz, Gavin W |
| Chinese Acad Sci / Chinese Academy of Sciences | Chinese Academy of Sciences |
| Houston Methodist Hosp / Houston Methodist Hospital | Houston Methodist Hospital |
| Imperial Coll London / Imperial College London | Imperial College London |
| Beijing Inst Technol / Beijing Institute of Technology | Beijing Institute of Technology |
| Shanghai Jiao Tong Univ / Shanghai Jiaotong University | Shanghai Jiao Tong University |
| Univ California San Diego / UC San Diego | University of California, San Diego |
| England / Scotland / Wales / Northern Ireland | United Kingdom |
| USA / U.S.A. / United States of America | United States |

# 7. Keyword cleaning and harmonization

Keyword cleaning was performed after record-level eligibility screening and was not used as a substitute for relevance assessment. Processing included lower-case comparison, punctuation/hyphen and whitespace standardization, singular/plural harmonization, synonym merging, within-document deduplication, and removal of demographic/indexing noise. The canonical display term retained conventional capitalization.

**Supplementary Table S6. Representative keyword harmonization and noise-removal rules.**

| **Original term(s)** | **Action / canonical term** |
| --- | --- |
| Robotic; Robotics; Robots | Merge as Robotics |
| Robot-assisted; Robot assisted; Robot-assisted surgery | Merge as Robot-assisted surgery |
| Catheter; Catheters | Merge as Catheter |
| Guide wire; Guide-wire; Guidewire | Merge as Guidewire |
| Endovascular intervention; Endovascular interventions | Merge as Endovascular intervention |
| Male; Female; Humans; Adult; Aged | Remove demographic/indexing noise |
| Article; Review; Controlled study; Priority journal | Remove publication/indexing noise |
| Human; Animal; Nonhuman | Remove species/indexing noise unless analytically required |
| Systematic review; Meta-analysis; PRISMA; Google Scholar | Remove generic reporting/method terms from topic-model text |

# 8. Software-ready data and import validation

Separate WoS and Scopus datasets were retained for database-specific analyses. The merged 541-record dataset was used only for analyses explicitly requiring a cross-database corpus, such as deduplicated topic modeling. All tagged files use a Web of Science plaintext structure that can be imported into CiteSpace, VOSviewer, and Bibliometrix/Biblioshiny.

**Supplementary Table S7. Final software-ready datasets.**

| **Dataset** | **Records** | **Intended use / validation** |
| --- | --- | --- |
| WoS cleaned tagged text | 373 | CiteSpace, VOSviewer, Biblioshiny; R import: 373/373 titles and years |
| Scopus converted tagged text | 493 | CiteSpace, VOSviewer, Biblioshiny; R import: 493/493 titles and years |
| Merged deduplicated tagged text | 541 | Combined VOSviewer/Biblioshiny/CiteSpace analyses and LDA; R import: 541/541 |
| Merged audit CSV | 541 | LDA text preparation, reproducibility, and record-level inspection |

Structural quality assurance confirmed PT = ER for every tagged file and one terminal EF marker per file. The Scopus conversion retained cited-reference fields for 484 records (18,371 references in total) and total citation counts for all 493 records.

# 9. Reproducibility and audit files

The complete screening master table records source database, source record number, DOI, title, bibliographic fields, final decision, exclusion code, exclusion reason, and adjudication basis. This permits independent record-level verification and prevents undocumented post hoc exclusions.

•	Supplementary Data S1: record-level screening audit covering all 690 WoS records and the 1,061 Scopus records retained after the combined document-type, language, and year restrictions. The 555 Scopus records removed by those metadata restrictions are reported at the aggregate level in Supplementary Table S1 and are not represented as individual rows in Supplementary Data S1.

•	Supplementary Data S2: final excluded records with one primary exclusion reason per record.

•	Supplementary Data S3: exact cross-database duplicate pairs.

•	Supplementary Data S4: internal duplicate removed from Scopus.

•	Supplementary Data S5: merged deduplicated 541-record audit CSV.

# 10. Analysis-specific counting and normalization

Productivity rankings for authors and institutions used fractional counting so that each publication contributed a total weight of one across its co-authors or affiliated institutions. Full counting was retained only for collaboration and co-occurrence network construction, where an edge represents the presence of a relation within a publication. Network link weights were normalized using association strength. Total citations and citations per year were reported separately to reduce, but not eliminate, citation-time bias. Rankings were interpreted as corpus-relative bibliometric indicators rather than as direct measures of overall scientific impact.

**Supplementary Table S8. Counting framework by analysis type.**

| **Analysis** | **Counting / normalization** | **Interpretive boundary** |
| --- | --- | --- |
| Author and institution productivity rankings | Fractional counting | Corpus-relative contribution |
| Country, author, institution, and keyword networks | Full counting; association-strength normalization | Relational structure, not individual impact |
| Citation tables | Total citations and citations per year | Database-specific; affected by citation window |
| Cross-database keyword matrix | Document-level binary co-occurrence | Each term or term pair counted at most once per document |

# 11. LDA corpus construction, model selection, stability, and trend analysis

The LDA source corpus comprised 541 deduplicated publications. English abstracts were available for 527 records. Fourteen records without abstracts and three records containing fewer than 20 retained tokens after preprocessing were excluded from model fitting, leaving 524 documents. Preprocessing comprised lower-casing; transliteration; removal of URLs, DOI strings, punctuation, numbers, English stop words, demographic/indexing terms, copyright text, and generic reporting language; normalization of recurrent terms and phrases; and construction of domain-aware unigrams and adjacent bigrams. Terms occurring in fewer than five documents or in more than 95% of abstract-bearing records were removed. The final document-term matrix contained 1,997 terms and 43,402 retained tokens.

Candidate topic numbers K = 3–10 were compared using perplexity, normalized UMass coherence calculated from the 15 highest-probability terms per topic, and repeated-seed stability measured as the mean Jaccard similarity of top-20 term sets after optimal topic alignment. K = 3 achieved the highest coherence and repeated-seed stability, whereas perplexity decreased monotonically as K increased. The five-topic solution was retained because it provided a more informative yet still parsimonious separation of the principal engineering and clinical domains; it was not interpreted as uniformly superior on every metric. Within K = 5, a 3 × 3 grid of alpha (0.01, 0.05, 0.10) and delta (0.005, 0.01, 0.05) selected alpha = 0.01 and delta = 0.05 by the combined rank of perplexity, coherence, and repeated-seed stability. The final Gibbs model used seed 20260902, burn-in = 1,500, iterations = 3,500, and thinning = 50.

**Supplementary Table S9. Comparison of candidate topic numbers using the selected K = 5 hyperparameter pair.**

| **K** | **Alpha/Delta** | **Perplexity** | **UMass coherence** | **Repeated-seed Jaccard** | **Combined rank sum** |
| --- | --- | --- | --- | --- | --- |
| 3 | 0.01/0.05 | 799.35 | −1.4622 | 0.8642 | 10 |
| 4 | 0.01/0.05 | 735.12 | −1.5084 | 0.6059 | 13 |
| 5 | 0.01/0.05 | 689.73 | −1.5705 | 0.5934 | 15 |
| 6 | 0.01/0.05 | 647.81 | −1.6046 | 0.6735 | 12 |
| 7 | 0.01/0.05 | 617.51 | −1.5904 | 0.6479 | 11 |
| 8 | 0.01/0.05 | 594.52 | −1.6119 | 0.5364 | 17 |
| 9 | 0.01/0.05 | 574.43 | −1.6115 | 0.5968 | 13 |
| 10 | 0.01/0.05 | 556.86 | −1.6144 | 0.5109 | 17 |

Repeated-seed fits used seeds 20260902, 20260919, and 20260936 with burn-in = 400, iterations = 800, and thinning = 40. Twenty bootstrap resamples used seeds 20260903–20260922 with burn-in = 300, iterations = 700, and thinning = 40, and were aligned to the final model by maximum-weight topic matching. Topic labels were assigned after reviewing LDAvis relevance-ranked terms (lambda = 0.60), high-probability terms, and representative documents. Annual values were calculated as the mean document-topic posterior probability among eligible publications in each year.

**Supplementary Table S10. Hyperparameter grid for the five-topic LDA model.**

| **Alpha** | **Delta** | **Perplexity** | **UMass coherence** | **Repeated-seed Jaccard** | **Rank sum** |
| --- | --- | --- | --- | --- | --- |
| 0.01 | 0.05 | 689.73 | −1.5705 | 0.5934 | 6 |
| 0.05 | 0.01 | 714.27 | −1.5985 | 0.6256 | 10 |
| 0.10 | 0.05 | 698.63 | −1.5878 | 0.5411 | 12 |
| 0.10 | 0.01 | 715.31 | −1.5944 | 0.5934 | 13 |
| 0.05 | 0.05 | 692.89 | −1.6477 | 0.6176 | 13 |
| 0.01 | 0.005 | 727.77 | −1.5666 | 0.4506 | 17 |
| 0.01 | 0.01 | 723.55 | −1.6017 | 0.4670 | 20 |
| 0.05 | 0.005 | 729.97 | −1.6248 | 0.5040 | 22 |
| 0.10 | 0.005 | 731.46 | −1.6343 | 0.5600 | 22 |

Supplementary Table S11. Five-topic labels, prevalence, and representative terms.

| **Thematic domain** | **Prevalence** | **Dominant documents** | **Selected representative terms** |
| --- | --- | --- | --- |
| Catheter teleoperation and haptics | 17.02% | 85 | catheter; force; sensor; haptic feedback; guidewire; control |
| Remote image-guided platforms | 30.12% | 154 | endovascular; navigation; imaging; tracking; remote; radiation |
| Magnetic soft/microrobots | 17.89% | 113 | magnetic actuation; soft robot; microrobot; steering; guidewire; MRI |
| Robotic PCI and outcomes | 23.02% | 115 | PCI; coronary; lesion; robotic PCI; operator; procedural outcome |
| Vascular surgery and thrombectomy | 11.94% | 57 | artery; embolization; aneurysm; aortic; thrombectomy; IVC |

*Note: Prevalence is the corpus-wide mean posterior topic probability among the 524 modeled documents. Dominant documents were assigned by the maximum document-topic posterior* probability. Labels are interpretive summaries and do not alter model estimation.

**Supplementary Table S12. Exploratory temporal trends in annual mean posterior topic prevalence.**

| **Topic** | **Thematic domain** | **Slope/year** | **95% CI lower** | **95% CI upper** | **P value** | **BH-adjusted P** | **R²** | **Interpretation** |
| --- | --- | --- | --- | --- | --- | --- | --- | --- |
| 1 | Catheter teleoperation and haptics | -0.00276 | -0.02115 | 0.01562 | 0.74174 | 0.74174 | 0.0127 | No significant linear trend |
| 2 | Remote image-guided platforms | 0.00995 | 0.00083 | 0.01907 | 0.03574 | 0.05957 | 0.4034 | No significant linear trend |
| 3 | Magnetic soft/microrobots | 0.01474 | 0.00743 | 0.02205 | 0.00136 | 0.00680 | 0.6982 | Rising |
| 4 | Robotic PCI and outcomes | -0.00397 | -0.01697 | 0.00902 | 0.50647 | 0.63308 | 0.0505 | No significant linear trend |
| 5 | Vascular surgery and thrombectomy | -0.01795 | -0.03211 | -0.00379 | 0.01854 | 0.04635 | 0.4775 | Declining |

*Note: Ordinary least-squares models used 11 annual observations (2015–2025). Because topic probabilities are compositional and annual publication counts differ, these results are interpreted as exploratory. Statistical significance was assessed using the Benjamini–Hochberg-adjusted P value.*

**Supplementary Table S13. Bootstrap stability of the final five-topic LDA model.**

| **Bootstrap resample** | **Aligned mean top-20 Jaccard** | **Bootstrap resample** | **Aligned mean top-20 Jaccard** |
| --- | --- | --- | --- |
| 1 | 0.4928 | 11 | 0.4701 |
| 2 | 0.4981 | 12 | 0.5036 |
| 3 | 0.5107 | 13 | 0.4772 |
| 4 | 0.5026 | 14 | 0.5535 |
| 5 | 0.5114 | 15 | 0.4867 |
| 6 | 0.4756 | 16 | 0.3544 |
| 7 | 0.5226 | 17 | 0.4855 |
| 8 | 0.5011 | 18 | 0.4283 |
| 9 | 0.5970 | 19 | 0.4916 |
| 10 | 0.4738 | 20 | 0.5755 |

*Note: Twenty document-level bootstrap resamples were aligned to the final model by maximum-weight topic matching. The aligned mean top-20-term Jaccard similarity was 0.4956 (SD 0.0502; range 0.3544–0.5970), indicating moderate rather than perfect topic-term stability.*

# 12. Database-source sensitivity analysis of the LDA solution

Because the primary LDA analysis used the deduplicated cross-database corpus, a database-source sensitivity analysis was performed to determine whether the five-topic structure was reproducible in WoSCC-only and Scopus-only corpora. These analyses evaluate sensitivity to database coverage and metadata harmonization; they are not presented as independent external validation because the two databases share 325 publications.

**Supplementary Table S14. Database overlap and LDA corpus construction.**

| **Corpus/component** | **Input records** | **Records with abstract** | **LDA-eligible records** | **Vocabulary terms** | **Retained tokens** |
| --- | --- | --- | --- | --- | --- |
| WoSCC-only | 373 | 369 | 365 | 1,546 | 29,881 |
| Scopus-only | 493 | 480 | 477 | 1,861 | 39,140 |
| Merged deduplicated corpus | 541 | 527 | 524 | 1,997 | 43,402 |
| Shared by both databases | 325 (60.1% of union) | – | – | – | – |
| WoSCC-unique | 48 (8.9% of union) | – | – | – | – |
| Scopus-unique | 168 (31.1% of union) | – | – | – | – |

*LDA eligibility required a non-empty English abstract and at least 20 retained tokens after preprocessing. Percentages for database overlap use the 541-record union as the denominator.*

**Supplementary Table S15. Fixed K = 5 model diagnostics and internal stability.**

| **Corpus** | **Perplexity** | **UMass coherence** | **Repeated-seed top-20 Jaccard** | **Bootstrap top-20 Jaccard, mean (SD)** |
| --- | --- | --- | --- | --- |
| WoSCC-only | 554.81 | −1.4890 | 0.7418 | 0.4498 (0.0566) |
| Scopus-only | 652.28 | −1.5494 | 0.5985 | 0.4733 (0.0590) |
| Merged corpus | 689.73 | −1.5705 | 0.5934 | 0.4956 (0.0502) |

All models used K = 5, alpha = 0.01, delta = 0.05, and the same Gibbs-sampling settings. Repeated-seed stability used three seeds; bootstrap stability used 20 document-level resamples. Perplexity values are corpus-dependent and should not be interpreted as direct evidence that one database model is superior to another.

**Supplementary Table S16. Topic-term reproducibility after alignment to the merged-corpus model.**

| **Merged-corpus topic** | **Selected merged terms** | **Matched WoSCC-only terms** | **Jaccard** | **Matched Scopus-only terms** | **Jaccard** |
| --- | --- | --- | --- | --- | --- |
| Catheter teleoperation and haptics | catheter; force; sensor; guidewire; control; haptic | catheter; force; sensor; haptic; control; guidewire | 0.818 | catheter; force; control; sensor; guidewire; haptic | 0.818 |
| Remote image-guided platforms | endovascular; navigation; imaging; tracking; remote; radiation | catheter; imaging; navigation; guidewire; tracking; vessel | 0.379 | endovascular; navigation; catheter; imaging; vessel; remote | 0.538 |
| Magnetic soft/microrobots | magnetic; guidewire; soft; actuation; microrobot; steering | magnetic; control; soft; navigation; guidewire; actuation | 0.600 | magnetic; guidewire; control; catheter; soft; actuation | 0.818 |
| Robotic PCI and outcomes | PCI; coronary; lesion; operator; robotic PCI; outcome | PCI; manual; procedural; lesion; coronary; operator | 0.600 | PCI; coronary; manual; lesion; procedural; operator | 0.818 |
| Vascular surgery and thrombectomy | artery; embolization; stent; aneurysm; aortic; thrombectomy | endovascular; artery; remote; navigation; treatment; catheter | 0.333 | artery; stent; renal; embolization; carotid; endovascular | 0.429 |

Topics were matched one-to-one using the Hungarian algorithm applied to pairwise Jaccard similarities of the top 20 probability-ranked terms. Mean matched similarity was 0.546 for WoSCC-only and 0.684 for Scopus-only. The selected terms shown here are abbreviated for readability; complete top-30 term lists are supplied as source data.

**Supplementary Figure S1. Cross-database reproducibility of the five-topic LDA solution.**

Cell values represent top-20 term Jaccard similarity between each database-specific topic and its optimally aligned merged-corpus topic. Higher values indicate greater term-set reproducibility.

**Supplementary Table S17. Temporal concordance of database-specific and merged-corpus topic prevalence.**

| **Topic** | **WoSCC slope** | **Scopus slope** | **Merged slope** | **rho: WoSCC vs merged** | **rho: Scopus vs merged** | **Interpretation** |
| --- | --- | --- | --- | --- | --- | --- |
| Catheter teleoperation and haptics | +0.0040 | −0.0029 | −0.0028 | 0.800 | 0.964 | Similar annual fluctuations; no significant linear trend |
| Remote image-guided platforms | −0.0189 | +0.0076 | +0.0099 | 0.045 | 0.773 | Direction and trajectory depend on database source |
| Magnetic soft/microrobots | +0.0247 | +0.0095 | +0.0147 | 0.900 | 0.836 | Consistent positive direction; strongest temporal reproducibility |
| Robotic PCI and outcomes | −0.0104 | −0.0048 | −0.0040 | 0.809 | 0.964 | Consistent weak negative direction; no significant adjusted trend |
| Vascular surgery and thrombectomy | +0.0005 | −0.0094 | −0.0180 | 0.345 | 0.873 | WoSCC trajectory differs; merged decline should be interpreted cautiously |

Slopes are changes in annual mean posterior probability per year. Spearman coefficients compare the 11 annual values from 2015–2025. Linear trends were evaluated descriptively with Benjamini–Hochberg correction within each corpus. The magnetic soft/microrobot slope was positive in all three corpora and statistically significant after Benjamini–Hochberg correction in WoSCC-only and merged analyses, but not in Scopus-only. The remote-platform decrease was significant only in WoSCC-only. The vascular surgery/thrombectomy decrease was not directionally concordant across all three corpora and is therefore interpreted as database-sensitive.

Lines show annual mean posterior topic probabilities from fixed K = 5 models fitted separately to WoSCC-only, Scopus-only, and merged deduplicated corpora. Curves are unsmoothed and are interpreted as descriptive, compositional trends.

Interpretation of database-source sensitivity

The sensitivity analysis supports moderate reproduction of the major technical and clinical domains, particularly catheter teleoperation and haptics, magnetic soft/microrobots, and robotic PCI. The lower similarities for remote image-guided platforms and vascular surgery/thrombectomy indicate that boundaries between these clinically oriented topics are affected by database coverage. Accordingly, the merged model is retained as the primary comprehensive synthesis, while claims about temporal emergence are restricted to patterns with concordant direction across corpora. The single-database analyses are therefore treated as sensitivity analyses rather than proof of complete database invariance.

# 13. Software environments, access dates, and reproducibility details

Bibliometric analyses and visual summaries: R 4.6.1 with Bibliometrix/Biblioshiny 5.4.1.

Network and burst analyses: VOSviewer 1.6.20 and CiteSpace 6.4.R1.

LDA modeling: R 4.6.1; topicmodels 0.2-17; tm 0.7-19; slam 0.1-56; and clue 0.3-68. The final models used Gibbs sampling.

Journal metrics: 2025 Journal Impact Factors were obtained from the 2026 edition of Clarivate Journal Citation Reports, accessed on September 3, 2026.

Supplementary Data S1–S5 are supplied as separate machine-readable audit files for peer review and reproducibility. Proprietary full-text content and the complete original database exports are not redistributed.

**Supplementary Table S18. VOSviewer network thresholds and final network sizes.**

| Analysis | Database | Minimum threshold | Nodes | Edges | Normalization |
| --- | --- | --- | --- | --- | --- |
| Country co-authorship | WoSCC | 5 documents | 17 | 56 | Association strength |
| Country co-authorship | Scopus | 5 documents | 22 | 110 | Association strength |
| Cited-source co-citation | WoSCC | 20 citations | 133 | 7,889 | Association strength |
| Cited-source co-citation | Scopus | 20 citations | 129 | 3,658 | Association strength |
| Author co-authorship | WoSCC | 5 documents | 10 | 25 | Association strength |
| Author co-authorship | Scopus | 5 documents | 17 | 35 | Association strength |
| Keyword co-occurrence | WoSCC | 5 occurrences | 118 | 1,876 | Association strength |
| Keyword co-occurrence | Scopus | 5 occurrences | 347 | 13,505 | Association strength |

Note: Edge counts were calculated from the final exported VOSviewer networks as one half of the summed node-level link counts. Full counting was used for collaboration and co-occurrence networks, and link weights were normalized by association strength. Productivity rankings for authors and institutions were calculated separately using fractional counting.

CiteSpace settings: publication years 2015–2025; one-year time slices; Top N = 50 per slice; burst-detection gamma = 1; minimum burst duration = 2 years; and Pathfinder plus pruning of sliced networks for network pruning.
